# m^6^A in mRNA coding regions promotes translation via the RNA helicase-containing YTHDC2

**DOI:** 10.1038/s41467-019-13317-9

**Published:** 2019-11-25

**Authors:** Yuanhui Mao, Leiming Dong, Xiao-Min Liu, Jiayin Guo, Honghui Ma, Bin Shen, Shu-Bing Qian

**Affiliations:** 1 Division of Nutritional Sciences, Cornell University, Ithaca, NY 14853 USA; 2 State Key Laboratory of Reproductive Medicine, Department of Histology and Embryology, Nanjing Medical University, Nanjing, 211166 China; 3 Key Laboratory of Arrhythmias of the Ministry of Education of China, East Hospital, Tongji University School of Medicine, Shanghai, 200120 China

**Keywords:** Biochemistry, Chemical biology, Genetics

## Abstract

Dynamic mRNA modification in the form of *N*^*6*^-methyladenosine (m^6^A) adds considerable richness and sophistication to gene regulation. The m^6^A mark is asymmetrically distributed along mature mRNAs, with approximately 35% of m^6^A residues located within the coding region (CDS). It has been suggested that methylation in CDS slows down translation elongation. However, neither the decoding feature of endogenous mRNAs nor the physiological significance of CDS m^6^A has been clearly defined. Here, we found that CDS m^6^A leads to ribosome pausing in a codon-specific manner. Unexpectedly, removing CDS m^6^A from these transcripts results in a further decrease of translation. A systemic analysis of RNA structural datasets revealed that CDS m^6^A positively regulates translation by resolving mRNA secondary structures. We further demonstrate that the elongation-promoting effect of CDS methylation requires the RNA helicase-containing m^6^A reader YTHDC2. Our findings established the physiological significance of CDS methylation and uncovered non-overlapping function of m^6^A reader proteins.

## Abstract (web summary)

Several functions have been proposed for m^6^A in RNA metabolism, yet little is known regarding the specific functions of individual m^6^A readers. Here, the authors observe that the m^6^A reader YTHDC2 — which contains an RNA helicase domain — acts on the coding region to promotes mRNA translation by resolving secondary structures.

## Introduction

A grand challenge in the postgenomic era is to elucidate complex layers of regulatory elements beyond the nucleotide sequence. mRNAs carry the genetic information that is translated by ribosomes. Both the 5′ and 3′ untranslated regions (UTRs) bear many *cis*-acting elements that are intricately linked to the regulation of translation initiation. The importance of the coding region (CDS) is apparent because the elongation speed directly controls the translational output. Recent findings from ribosome profiling studies show that the translation machinery proceeds not at a constant rate but rather in a stop-and-go traffic manner^1,2^. Frequent ribosomal pausing decreases the overall translation efficiency (TE) by reducing the elongation speed and limiting the amount of free ribosomes available for other protein synthesis. Factors contributing to ribosomal pausing are likely to be multifaceted. Besides the nucleotide sequence, the flexible nature of mRNA molecules implies that particular shape can also encode regulatory information guiding translational control^3^. One fundamental question is how cells fine-tune the TE for individual transcripts by integrating parallel codes embedded within the nucleotide sequence.

One such parallel code is the chemical modification of nucleotides within mRNAs^4^. To date, more than 150 distinct modifications have been identified on RNA species^5^. *N*^6^-methyladenosine (m^6^A) is the most abundant internal base modification occurring on eukaryotic mRNAs. The m^6^A content varies substantially across various species, tissues, and cellular environments^6^, suggesting an extensive regulation of methylation dynamics. The m^6^A topology is achieved by two opposing enzyme systems: the methyltransferase complex comprising a core heterodimer of METTL3–METTL14 (refs ^7,8^), and m^6^A demethylases FTO and ALKBH5 (refs ^9,10^). The biological effect of m^6^A largely depends on m^6^A reader proteins, such as YTH domain-containing proteins^11^. A recent study reported that m^6^A also repels certain RNA-binding proteins^12^, forming an additional layer in controlling dynamic RNA–protein interaction. By affecting nearly all the aspects of mRNA metabolism, m^6^A marks an ever growing list of cellular and physiological functions.

Despite the tremendous progress in the functional characterization of m^6^A modification, the regional effects of mRNA methylation remain obscure. For mature mRNAs, there is a strong enrichment of m^6^A around the stop codon and 3′ UTR^13,14^. The asymmetric m^6^A deposition suggests that regional methylation may have distinct functional consequences. Previous studies reported that the cytosolic m^6^A readers YTHDF1 and YTHDF3 promote cap-dependent mRNA translation presumably via 3′ UTR methylation^15,16^. Recent m^6^A-seq studies also revealed m^6^A peaks in 5′ UTR and start codons when plotting the peak density along the transcriptome^17^. Intriguingly, m^6^A in the 5′ UTR could facilitate cap-independent translation through a process involving eIF3 (ref. ^18^), although the exact nature of this process remains unclear. Besides m^6^A in the untranslated regions, approximately 35% of m^6^A residues are located within the CDS. Using an elegant single molecule-based in vitro translation system, it has been demonstrated that m^6^A interferes with the decoding process by affecting tRNA accommodation, thereby slowing down translation elongation^19^. However, neither the decoding feature of methylated codons within endogenous mRNAs nor the physiological significance of CDS methylation has been clearly defined.

Given the forward movement of ribosome during elongation, the CDS methylation could impede the translation by directly affecting the decoding process or indirectly blocking elongation via m^6^A-binding proteins. Acting as brakes and roadblocks, these mechanisms are expected to result in prominent ribosome pausing. Such persistent ribosome stalling is expected to trigger the mRNA surveillance system and subsequent mRNA degradation. However, the logical and mechanistic relationships between CDS methylation and ribosome dynamics are poorly understood, if such relationships exist. Here we set out to determine the relationship between CDS m^6^A modification and ribosome behavior. Unexpectedly, we found that CDS methylation positively regulates translation by resolving mRNA secondary structures. Intriguingly, the translation-promoting effect of m^6^A modification requires YTHDC2, the only RNA helicase-containing m^6^A reader. Our findings establish the physiological significance of m^6^A methylation in CDS and the unique role of YTHDC2 in translation elongation suggests nonoverlapping functions of m^6^A reader proteins.

## Results

### CDS m^6^A occurs on mRNAs with low TE

**Fig. 1:**
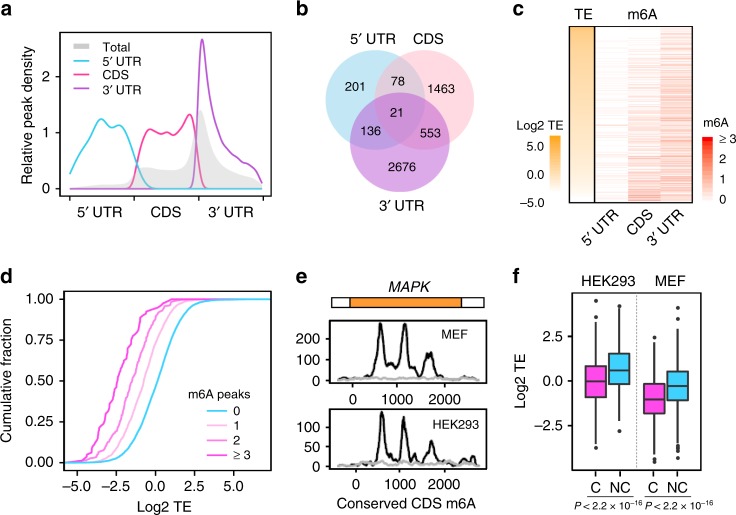
CDS m^6^A is enriched in transcripts with inactive translation. **a** Distribution of m^6^A sites along transcripts with different regional methylation. Transcripts are grouped into 5′ UTR, CDS, and 3′ UTR methylation based on the identified m^6^A sites. Only METTL3 or WTAP sensitive m^6^A sites are considered. The distribution of total m^6^A sites was shown in gray. UTR: untranslated region, CDS: protein coding region. **b** A Venn diagram shows the overlapping of transcripts containing m^6^A sites in different regions. **c** Translation efficiency (TE, left column) and m^6^A density across the transcriptome are presented as parallel heat-maps. Note the inverse correlation between CDS m^6^A methylation and TE. **d** Translation efficiency (TE) is plotted as accumulative fractions for mRNAs with different amount of m^6^A peaks in CDS. **e** A representative example (*MAPK*) of evolutionary conserved m^6^A sites between MEF and HEK293 cells. The coverage of m^6^A is shown as black lines and the input as gray. **f** A box plot shows the translation efficiency (TE) of mRNAs containing conserved (C) and non-conserved (NC) m^6^A sites in MEF and HEK293 cells (Wilcox test, all *P* values < 2.2 × 10^−^^16^). The median of TE in each group is indicated by a center line, the box shows the upper and lower quantiles, whiskers shows the 1.5× interquartile range, and the outliers are indicated by points. Source data are provided as a Source Data file.

We began our analyses by identifying m^6^A peaks from mouse embryonic fibroblasts (MEF) using a method described before^20^. To avoid false positives due to background noise and possible bias of peak calling, only METTL3 or WTAP sensitive m^6^A peaks are used in the following analysis. From a total of 15,646 m^6^A peaks identified from MEF cells, only 8352 peaks show >50% decrease of methylation upon depletion of METTL3 or WTAP. As expected, these m^6^A peaks are mostly enriched near the stop codon (Fig. 1a, gray line). Based on their positions within mRNA, we classified these m^6^A sites into three regions: 5′ UTR (449 peaks from 436 genes), CDS (2825 peaks from 2115 genes) and 3′ UTR (4044 peaks from 3386 genes). Notably, more than half of the transcripts contain one regional m^6^A (Fig. 1b). Only a few messengers harbor m^6^A in all three regions. To reveal possible relationships between regional methylation and gene functions, we searched for common biological themes among transcripts bearing m^6^A in different regions. Interestingly, genes involved in transcriptional regulation are overrepresented among mRNAs with CDS methylation (Supplementary Fig. 1).

Given the crucial role of CDS in translation elongation, we next sought to determine the correlation between CDS methylation and TE. Using ribosome profiling (Ribo-seq) data sets obtained from MEF cells, we computed TE by normalizing ribosome density with the corresponding mRNA levels. Notably, neither 5′ UTR nor 3′ UTR methylation correlates with TE in a significant manner (Fig. 1c). Only when CDS methylation is considered, does an inverse correlation become evident between m^6^A levels and TE. In comparison to the non-methylated mRNAs, transcripts with CDS methylation show significantly reduced ribosome occupancy (Wilcox-test, *P* < 2.2 × 10^−16^, Supplementary Fig. 2a). Remarkably, as the number of CDS m^6^A peaks increases, the ribosome occupancy decreases correspondingly with mRNAs bearing >3 m^6^A peaks exhibiting the lowest TE (Fig. 1d). The similar finding was observed by analyzing CDS m^6^A coverage (Supplementary Fig. 2b), which is further corroborated by the distribution of TE for transcripts with or without CDS m^6^A (Supplementary Fig. 2c). The negative correlation between CDS methylation and ribosome occupancy also holds true in a human cell line HEK293 (Supplementary Fig. 2d), as well as mouse embryonic stem cells (ESCs) and embryonic bodies (EBs)^21^ (Supplementary Fig. 2e). Since the calculated TE is normalized by mRNA levels, the inverse correlation between TE and CDS methylation cannot be explained by m^6^A-mediated mRNA degradation. It is rather consistent with the notion that CDS methylation occurs on transcripts with relatively inactive translation.

We noticed that certain m^6^A peaks in CDS are highly reproducible in different cell lines, suggesting a functional conservation. One typical example is *MAPK7* that bears three prominent CDS m^6^A peaks (Fig. 1e). Using m^6^A-seq data sets obtained from human (HEK293) and mouse (MEF) cells, we identified approximately 10% of m^6^A peaks (429 peaks of 316 mRNAs) as the conserved methylation sites in CDS. Notably, transcripts harboring the conserved m^6^A sites exhibit significantly lower ribosome occupancy than ones containing the non-conserved sites (Fig. 1f). Therefore, CDS methylation could be evolved and retained on certain transcripts with functional significance.

### CDS m^6^A methylation leads to ribosome pausing

**Fig. 2:**
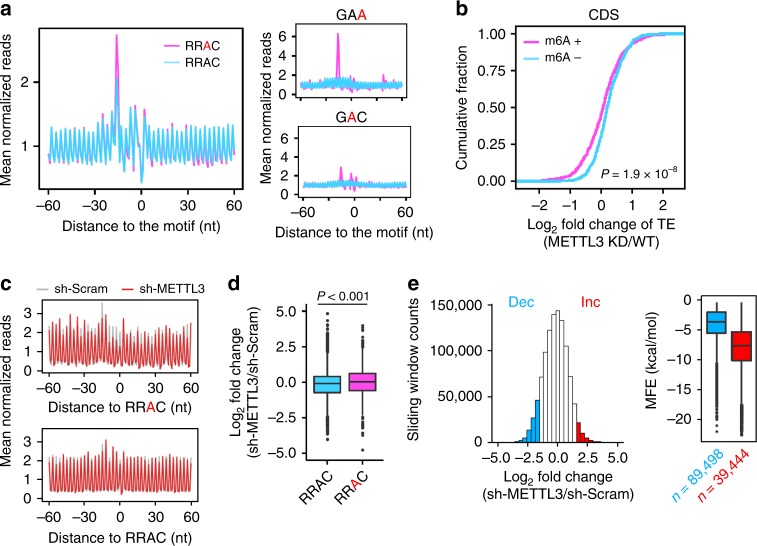
Characterize the role of CDS m^6^A in translation efficiency. **a** Aggregation plots show the mean ribosome densities along mRNA regions aligned to the RRAC motif with (pink line) or without (blue line) m^6^A modification. Right panels show the mean ribosome densities along mRNA regions aligned to GAA or GAC codons with (pink line) or without (blue line) m^6^A modification. A minus position value indicates upstream of m^6^A sites, whereas a positive value indicates downstream of m^6^A sites. The m^6^A site in RRAC motif or codons is highlighted by red. **b** The fold change of translation efficiency upon METTL3 knockdown is plotted as accumulative fractions (Wilcox test, *P* = 1.9 × 10^−^^8^) for mRNAs bearing CDS methylation (m^6^A+) or not (m^6^A−). Both groups have similar levels of basal TE. **c** Aggregation plots show the mean ribosome densities along mRNA regions aligned to the RRAC motif with (top panel) or without (bottom panel) m^6^A modification. Plots from cells with or without METTL3 knockdown are color coded. The m^6^A site in RRAC motif is highlighted by red. **d** From the same data sets as **c**, the fold change of ribosome accumulation upstream of the motif in response to METTL3 knockdown is calculated for transcripts with or without m^6^A modification (Wilcox test, *P* < 0.001). For the boxplot in **d** and **e**, the median value in each group is indicated by a center line, the box shows the upper and lower quantiles, whiskers shows the 1.5× interquartile range, and the outliers are indicated by points. **e** A histogram shows the distribution of changes of regional ribosome density in response to METTL3 knockdown. A sliding window of 30 nt in length with a step of 3 nt are used to calculate the local ribosome density. Regions with <1/3-fold (Dec, blue) and >3-fold (Inc., red) changes are highlighted by color coding. The right box plot shows the predicted minimum folding free energy (MFE) for regions with <1/3-fold (Dec) and >3-fold (Inc.) changes (Wilcox test, *P* < 2.2 × 10^−16^). Source data are provided as a Source Data file.

A previous study demonstrated that presence of m^6^A interferes with the decoding process of ribosomes by affecting tRNA accommodation at the A site, at least in the in vitro translation system reconstituted from *Escherichia coli*^19^. We then examined whether CDS m^6^A methylation leads to ribosome pausing at specific mRNA positions in mammalian cells. We took advantage of single nucleotide resolution m^6^A sites identified from HEK293 cells^22^. In addition, we used Ribo-seq data sets obtained in HEK293 without cycloheximide pretreatment to avoid technical artifacts^23^. When transcripts are aligned to the identified m^6^A site, we observed an elevated ribosome density at the −15 nt position when the 5′ end of reads are counted (Fig. 2a). This position corresponds to the methylated codon at the ribosomal A site. The approximately threefold higher ribosome density when the A site codon is methylated suggests a delayed codon:anti-codon interaction in the presence of m^6^A. The similar result was also seen in MEFs (Supplementary Fig. 3a), although the low resolution m^6^A mapping possibly underestimates the m^6^A-induced ribosome pausing in these cells. Our analysis provides an in vivo evidence that CDS m^6^A methylation affects the decoding process of endogenous transcripts.

When the methylated codon enters the A site, the m^6^A could be in any positions of the triplet. Based on the consensus sequence of m^6^A (Supplementary Fig. 3b), we calculated the ribosome occupancy for different types of methylated codons. Notably, G**A**C (**A** as the methylated adenosine) accounts for the majority of methylated codons (41%). However, only a modest increase (approximately twofold) of ribosome pausing was observed when the methylated G**A**C triplet occupies the A site (Fig. 2a, right panel). Intriguingly, the less abundant GA**A** codon (10%) exhibited the strongest ribosome pausing among all the methylated codons. This is not due to the wobble position because the same m^6^A position in both AG**A** and GG**A** codons is only associated with a slight increase of ribosome density (Supplementary Fig. 3c). This finding suggests a strong codon-specific effect of m^6^A on ribosome dynamics.

### CDS m^6^A methylation promotes TE

The m^6^A-caused delay in the decoding process potentially explains the negative correlation between CDS methylation and the TE. However, correlation does not imply causation. If reduced TE is a direct consequence of CDS methylation, removal of m^6^A modification from CDS is expected to increase the translational output. To test this possibility, we knocked down the core m^6^A methyltransferase METTL3 from MEF cells using shRNA. As reported previously^24^, METTL3 knockdown resulted in nearly 30% decrease of global protein synthesis as determined by [^35^S] metabolic labeling (Supplementary Fig. 4a). Since METTL3 knockdown reduces mRNA methylation in a non-specific manner, it is possible that the effect of CDS methylation is masked by m^6^A in other regions. We therefore stratified mRNAs based on regional methylation followed by comparison of TE fold changes before and after METTL3 knockdown. While altering 3′ UTR methylation has modest effects on TE, reducing 5′ UTR m^6^A levels decreased ribosome occupancy (Supplementary Fig. 4b). This is consistent with previous findings about the potential role of 5′ UTR m^6^A methylation in non-canonical mRNA translation^18,24^. To our surprise, depletion of CDS methylation as a result of METTL3 knockdown also led to reduced TE (Fig. 2b). The same feature holds true in a human cell line HeLa (Supplementary Fig. 4c). In addition, we conducted the same analysis using published data sets obtained from EBs and ESCs. It is clear that transcripts bearing CDS methylation were more sensitive to METTL3 depletion than the m^6^A negative control by decreasing TE (Supplementary Fig. 4d).

The positive role of CDS methylation in translation is seemingly contradictory to the m^6^A-induced ribosomal pausing effect. We next examined whether the removal of m^6^A from CDS would eliminate ribosomal pausing. While MEF cells with scramble shRNA control showed a prominent ribosome pausing at the methylated A site (Fig. 2c, gray line), the elevated peak at the same site was largely diminished in MEFs lacking METTL3 (Fig. 2c, red line). Despite the lack of prominent pausing peaks, we observed an increased ribosome density upstream of the m^6^A site relative to the downstream region. This was not seen at the RRAC site without methylation (Fig. 2c, bottom panel). To achieve a more quantitative analysis, we computed the ratio of ribosome density in regions before and after the CDS m^6^A sites. Upon METTL3 knockdown, there was a significant increase of ribosome density in regions upstream of the m^6^A site (Fig. 2d). Using sliding window analysis (Supplementary Fig. 5a), we identified regional ribosome pausing induced by METTL3 knockdown across the whole transcriptome. Intriguingly, the regions with pausing sites caused by the lack of CDS methylation tend to form stable secondary structures (low minimum folding energy, MFE) (Fig. 2e). To substantiate this finding further, we repeated the same analysis using cells lacking ALKBH5, a m^6^A demethylase. Under the elevated m^6^A levels, we observed fewer pausing regions, and less structural features in pausing regions. (Supplementary Fig. 5b). Collectively, these results suggest that CDS methylation promotes TE by reducing ribosomal pausing, probably involving m^6^A-mediated structural switches.

### CDS m^6^A are associated with relaxed mRNA structures

**Fig. 3:**
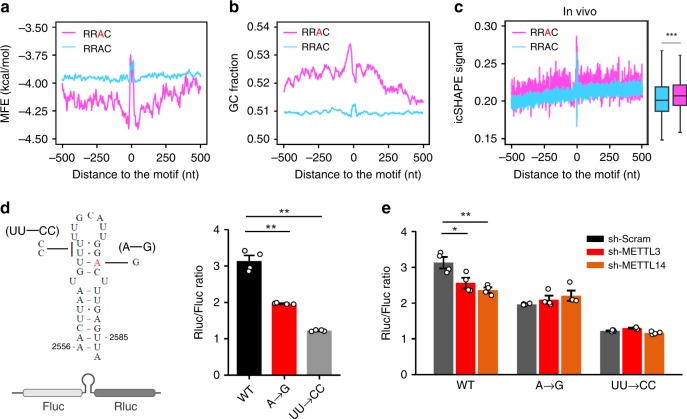
CDS m^6^A methylation resolves mRNA secondary structures. **a** The predicted minimum folding free energy (MFE) is plotted along mRNA regions surrounding the CDS RRAC motif with (pink line) or without (blue line) m^6^A modification. A sliding window with 30 nt in length and a step of 3 nt was used to calculate MFE. For each window, the central position is used for alignment. A minus position value indicates upstream of m^6^A sites, whereas a positive value indicates downstream of m^6^A sites. The m^6^A site in RRAC motif is highlighted by red. Notably, a lower MFE value indicates a higher potential for RNA secondary structures. **b** The GC content is plotted along mRNA regions surrounding the CDS RRAC motif with (pink line) or without (blue line) m^6^A modification. **c** The in vivo icSHAPE signal is plotted along mRNA regions surrounding the CDS RRAC motif with (pink) or without (blue) m^6^A modification. Notably, a higher in vivo icSHAPE signal indicates a less structured region. The right boxplot shows the average of icSHAPE signals across mRNA regions from –500 nt to 500 nt relative to the RRAC motif with (pink) or without (blue) m^6^A modification (Wilcox test, ***P < 0.001). The median of icSHAPE signals in each group is indicated by a center line, the box shows the upper and lower quantiles, whiskers shows the 1.5× interquartile range. The outliers are not shown. **d** The left panel shows the schematic of a dual luciferase reporter with a sandwiched secondary structure derived from *MALAT1* (2556–2587). Both UU → CC and A → G mutants are also shown. The m^6^A site is highlighted by red. The right panel shows the ratio of Rluc/Fluc in transfected cells expressing wild type or indicated mutants. Error bars, mean ± s.e.m.; Single-tailed *t* test, *n* = 4, *P < 0.05, **P < 0.01. **e** The ratio of Rluc/Fluc in transfected cells expressing wild type or mutant reporters, with either METTL3 or METTL14 knockdown. Error bars, mean ± s.e.m.; single-tailed *t* test, *n* = 4, *P < 0.05, **P < 0.01. Source data are provided as a Source Data file.

Previous studies demonstrated that m^6^A influences mRNA folding by acting as a structural switch^25^. In particular, m^6^A installation destabilizes RNA secondary structures^26^. To explore the mechanism explaining the positive role of CDS m^6^A in translation, we first analyzed the structural potential predicted by ViennaRNA in regions with or without m^6^A modification. From the mouse transcriptome, the methylated region displays a greater potential for stable secondary structures than the nonmethylated counterpart (Fig. 3a and Supplementary Fig. 6a). Consistently, analysis of the sequence context in methylated regions revealed a significant increase of the G/C content (Fig. 3b, Wilcox test, *P* < 2.2 × 10^–16^). These sequence-based structural prediction clearly indicates that m^6^A methylation sites are preferentially deposited to the regions tending to form stable structures. To probe the mRNA folding status in vivo, we took advantage of the icSHAPE data sets derived from MEF cells^27^. Unexpectedly, the methylated region shows higher icSHAPE signals than the non-methylated counterpart (Wilcox test, *P* < 0.001) (Fig. 3c), an indication of more single-stranded signals. The discrepancy between in vitro and in vivo structures was further confirmed by comparison of Gini index of methylated and non-methylated regions (Supplementary Fig. 6b, c). These results suggest that m^6^A modification in CDS likely eliminates local structures despite the relatively high GC content.

As an independent validation, we repeated our analyses using the available structural and m^6^A data sets derived from HepG2 cells. Once again, methylated CDS regions in human transcriptome have a stronger tendency of forming stable secondary structures as evidenced by lower MFE and higher GC content (Supplementary Fig. 6d, e). However, there was a clear decrease of PARS scores in mRNA regions containing CDS m^6^A modification (Supplementary Fig. 6f), an indication of increased single-strand RNA signals. Therefore, CDS methylation in the form of m^6^A acts to prevent the formation of stable secondary structures.

### CDS m^6^A modification resolves mRNA secondary structures

As ribosome occupancy can be influenced by both initiation and elongation, potential pausing sites in CDS could increase the ribosome density with reduced translational output. To directly demonstrate the critical role of m^6^A in CDS structures and subsequent translational outcomes, we constructed a fusion reporter by inserting a structural motif between firefly luciferase (Fluc) and renilla luciferase (Rluc) (Fig. 3d). The structural motif is derived from the non-coding RNA *MALAT1* ranging from 2556 to 2586 nt (ENST00000534336.1), which includes a well-characterized m^6^A site at position 2577. The resultant fusion protein contains an extra 20 amino acids between Fluc and Rluc (see Supplementary Methods). It has been demonstrated that m^6^A modification at the site of A2577 destabilizes RNA folding^25^, which enables us to investigate the effect of RNA structural changes in translation. To evaluate the effect of m^6^A-induced structural switch, we first created a mutant by replacing the nucleotide A at position 2577 with G (A → G), which abolishes methylation at this site. Consistent with the more stable stem loop structure in the absence of m^6^A, we observed a significant decrease (~35%) of downstream Rluc translation when compared to the wild type (Fig. 3d). Next, we mutated the dinucleotides UU at positon 2566 and 2567 to CC (UU → CC), which makes the structure more stable than wildtype by changing the two G•U base pairs to G•C. Indeed, we observed a further decrease of Rluc translation from this mutant (~63%) (Fig. 3d). Therefore, the secondary structure in CDS acts as a roadblock for elongating ribosomes.

The decreased Rluc translation in the A → G mutant supports the positive role for m^6^A in translation. To test the possibility that CDS m^6^A methylation promotes translation by resolving mRNA secondary structures, we examined the Rluc/Fluc ratio in cells lacking m^6^A methyltransferases. In cells with METTL3 or METTL14 knockdown, we observed 15% and 18% reduction of Rluc translation, respectively (Fig. 3e). This was not due to the pleiotropic effects of m^6^A writers because the same cells exhibited little decrease of Rluc translation for the A → G mutant. The methylation status of these reporters was confirmed using SELECT, a site-specific m^6^A detection method^28^ (Supplementary Fig. 7a). The UU → CC mutant largely diminishes the effect of m^6^A-dependent structural rearrangement^25^. As a result, this mutant is resistant to METTL3 or METTL14 knockdown (Fig. 3e).

To substantiate this finding further, we knocked down m^6^A demethylases ALKBH5 or FTO. Although both A → G and UU → CC mutants maintained the similar Rluc/Fluc ratio, we observed modest, but significant, increase of Rluc translation (Supplementary Fig. 7b). The modest effect is presumably due to the high basal levels of methylation (60–80%) at the position of 2577 (ref. ^6^), leaving limited room for further increase of m^6^A. Collectively, we conclude that m^6^A modification in the CDS, in spite of causing codon-specific ribosome pausing, promotes the overall TE by resolving stable secondary structures.

### YTHDC2 promotes TE

mRNA structures in CDS are commonly believed to be unwound by elongating ribosomes^29^. In line with this notion, transcriptome-wide structural mapping revealed that RNAs tend to assume more secondary structures in vitro than in vivo^27,30^. Since CDS methylation is enriched in transcripts with relatively inactive translation, one interesting question is whether m^6^A modification itself is able to resolve mRNA secondary structures. If so, a similar folding status of methylated regions is expected between in vitro and in vivo. However, this is not the case. Although the methylated regions showed increased in vivo icSHAPE signals (more single strand of RNA, Fig. 3c), the same region exhibited little difference of icSHAPE signals obtained in vitro when compared to the nonmethylated counterpart (Supplementary Fig. 7c). In addition, mRNAs with CDS methylation showed decreased in vivo icSHAPE signals upon METTL3 depletion (Supplementary Fig. 7d). This result argues that m^6^A modification relies on other factors inside cells in order to effectively unfold stable CDS structures.

**Fig. 4:**
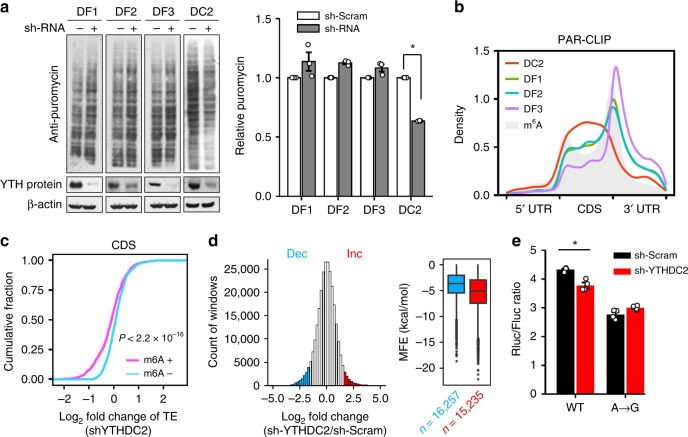
YTHDC2 promotes translation efficiency by acting on CDS m^6^A. **a** Puromycin labeling assay shows the global protein synthesis in HEK293 cells lacking each individual cytoplasmic m^6^A reader proteins. The right panel shows the quantitative results based on puromycin signals normalized with β-actin. Error bars, mean ± s.e.m.; *t* est, *n* = 3, *P < 0.05. DF1: YTHDF1, DF2: YTHDF2, DF3: YTHDF3, DC2: YTHDC2. **b** Distribution of the binding sites of cytoplasmic m^6^A reader proteins across the human transcriptome. All binding sites are identified from PAR-CLIP data sets obtained from HeLa cells. The distribution of m^6^A sites is shown as gray. **c** The fold change of translation efficiency upon YTHDC2 knockdown is plotted as accumulative fractions (Wilcox test, *P* < 2.2 × 10^−16^) for transcripts bearing CDS methylation (m^6^A+) or not (m^6^A−). Both groups have similar levels of basal TE. **d** A histogram shows the distribution of changes of regional ribosome density in response to YTHDC2 knockdown. A sliding window of 30 nt in length with a step of 3 nt are used to calculate the local ribosome density. Regions with <1/3-fold (Dec, blue) and >3-fold (Inc., red) changes are highlighted by color coding. The right box plot shows the predicted minimum folding free energy (MFE) for regions with <1/3-fold (Dec) and >3-fold (Inc.) changes (Wilcox test, *P* < 2.2 × 10^−16^). The median of MFE in each group is indicated by a center line, the box shows the upper and lower quantiles, whiskers shows the 1.5× interquartile range, and the outliers are indicated by points. **e** The ratio of Rluc/Fluc in transfected cells expressing wild type or mutant reporters, with or without YTHDC2 knockdown. Error bars, mean ± s.e.m.; Single-tailed *t* test, *n* = 4, *P < 0.05. Source data are provided as a Source Data file.

To search for potential m^6^A-interacting proteins that are capable of resolving CDS structures, we examined all the cytoplasmic YTH domain-containing m^6^A reader proteins. YTHDF1, YTHDF2, and YTHDF3 have been suggested to function cooperatively in the cytoplasm to promote efficient translation and degradation of specific m^6^A-containing mRNAs^15,16,31^. The multi-domain m^6^A reader YTHDC2 has been demonstrated to have an ATP-dependent RNA helicase activity^32,33^. If any of these m^6^A readers are actively participated in CDS methylation-promoted translation, silencing the corresponding genes would result in reduced translational output. We knocked down each m^6^A readers from HEK293 cells followed by measurement of global protein synthesis. Puromycin labeling of nascent polypeptides revealed that knocking down YTHDF1-3 had minimal effect on the global scale of mRNA translation (Fig. 4a). Remarkably, knocking down YTHDC2 led to a 40% reduction of protein synthesis. This is consistent with the observation that silencing YTHDC2, but not other m^6^A readers, significantly decreased the cell growth rate (Supplementary Fig. 8a). The translational effect of YTHDC2 is further corroborated by the polysome profiling in cells lacking YTHDC2 (Supplementary Fig. 8b). The sustained polysome in the absence of YTHDC2 is an indication of elongation pausing. Although YTHDC2 is highly expressed in testes cells (Supplementary Fig. 8c), it is clear that depleting this m^6^A reader readily affects protein synthesis even in cultured cells.

### YTHDC2 resolves mRNA secondary structures

YTHDC2 contains multiple domains that bind to RNAs with distinct nucleotide preferences^34^. By reanalyzing the PAR-CLIP data sets obtained from HeLa cells, we found a strong enrichment of YTHDC2 binding sites in the CDS, rather than the 3′ UTR where the other cytoplasmic m^6^A readers are enriched (Fig. 4b). In addition, these binding sites have increased m^6^A coverage relative to the random sequences (Supplementary Fig. 8d). To substantiate this finding further, we conducted PAR-CLIP using MCF7 cells transfected with Flag-tagged YTHDC2 (Supplementary Fig. 8e). It is clear that the majority of YTHDC2 binding sites are enriched in CDS. Recent studies reported that YTHDC2 directly interacts with the small ribosomal subunit^35^, which suggests the involvement of YTHDC2 in translation of certain transcripts^36–38^. Notably, YTHDC2 has several motifs that are characteristic of the DEAH/RNA helicase A family, implying a functional connection with RNA structures. To investigate whether YTHDC2 helps resolve mRNA structures marked by m^6^A, we conducted Ribo-seq in HEK293 cells with or without YTHDC2 knockdown. By comparing the TE of transcripts with or without CDS methylation, we found that mRNAs bearing methylated CDS are more sensitive to YTHDC2 depletion than ones with 3′ UTR methylation (Fig. 4c). Notably, YTHDC2 knockdown has little effect on the translation of mRNAs with 5′ UTR methylation (Supplementary Fig. 8f), confirming the regional effect of YTHDC2.

We next examined whether YTHDC2 depletion reduces translation by inducing ribosomal pausing. Indeed, regions with increased ribosome density in the absence of YTHDC2 exhibit stronger structural features (lower MFE, Fig. 4d). Finally, we assessed the role of YTHDC2 in the translation of structural reporters. Much like METTL3 knockdown (Fig. 3e), silencing YTHDC2 decreases the ratio of Rluc/Fluc (Fig. 4e). Importantly, the A → G mutant maintained the Rluc/Fluc ratio, suggesting that YTHDC2 facilitates ribosome movement over the structural hurdle in an m^6^A-dependent manner. To confirm the critical role of helicase activity, we conducted a rescue experiment by introducing an E332Q mutation to inactivate the helicase of YTHDC2 (ref. ^33^) (Supplementary Fig. 9a). Luciferase reporter assay showed that only the wild type YTHDC2, but not the helicase-dead mutant, was able to restore the Rluc/Fluc ratio (Supplementary Fig. 9b). Taken together, our results indicate that m^6^A modification in CDS promotes translation of structured mRNA by recruiting the RNA helicase-containing YTHDC2.

## Discussion

Recent studies have uncovered multiple roles of m^6^A in regulating translation. However, both positive and negative influences of this epitranscriptomic mark on protein production has been reported. Since methylation at different mRNA regions may have distinct functions, it is important to dissect regional effects of m^6^A on translation. In this regard, it is intriguing that approximately 35% of m^6^A residues are located within the CDS. Besides the embedded coding information, modified nucleotides like m^6^A could confer additional layer of regulation for the decoding process. Indeed, single molecule analysis of ribosome dynamics using an in vitro translation system derived from *E. coli* revealed that the presence of m^6^A in the CDS delays translation elongation^19^. Using synthetic mRNAs, random incorporation of m^6^A in general reduces translational output in transfected cells^17^. Consistently, for endogenous transcripts, we found that the greater the amount of m^6^A in CDS, the lower the TE. In addition, we demonstrated that methylated codons lead to pausing of elongating ribosomes, although the m^6^A is clearly not the sole determinant. The seemingly negative role of CDS methylation in the form of m^6^A in translation raises an intriguing question: what is the evolutionary benefit by creating potential roadblocks on endogenous transcripts?

To our surprise, removing CDS m^6^A from methylated transcripts did not result in enhanced translation. In fact, it further reduces the TE at least for some transcripts. This counter-intuitive result serves as a warning against attaching direct causative effects to what are merely suggestive correlations. Since the negative correlation is based on a pool of mRNAs with differential methylation, validating the causative effect requires examination of individual transcripts. Notably, the majority of methylated mRNAs only contain one reliable m^6^A peak and the density of CDS methylation on average is lower than that in 3′ UTR. Intriguingly, CDS m^6^A residues are usually located in regions that tend to form relatively stable secondary structures. This feature provides a plausible explanation why CDS m^6^A is highly enriched in mRNAs with less active translation. However, in the absence of METTL3, we observed newly formed ribosome pausing in mRNAs with methylated CDS. These results suggest that m^6^A installation is favorable for translation of structured mRNAs. For unstructured mRNAs with active translation, m^6^A modification likely reduces the TE by acting as a resistor. For structured mRNAs with poor TE, additional m^6^A marks could serve as a transconductor, facilitating translation elongation by resolving stable structures. How the structural region is selected for methylation is not known. It is possible that typical RNA structures may provide additional signals for recognition by m^6^A methyltransferases.

A major mechanism through which m^6^A regulates the fate of mRNAs is by recruiting m^6^A reader proteins^4^. Both YTHDF1 and YTHDF3 have been shown to mediate translational effects of m^6^A, mainly at the UTR regions^15,16^. It remains unclear how these reader proteins preferentially recognize the m^6^A mark in 3′ UTR, but not CDS. YTHDC2 is the largest YTH domain-containing protein and the only member of the family to contain helicase domains. YTHDC2 is highly expressed in testis and several recent studies demonstrated its crucial role in spermatogenesis^32,33,36,37^. It will be interesting to determine whether the transcriptome in germ cells tend to have more stable secondary structures. Regardless, many other human tissues express YTHDC2 at substantial levels, suggesting that the function of YTHDC2 is not limited to germ cells. Akin to the tissue-specific expression of YTHDC2, different cell types have varied subcellular localizations of m^6^A readers (Supplementary Fig. 10). Therefore, the functionality of m^6^A reader is likely context-dependent.

Unlike other m^6^A readers, silencing YTHDC2 from cells in culture resulted in a severe growth defect, arguing for a crucial role for YTHDC2 in cell physiology. Recent studies indicate that YTHDC2 increases the TE of a small subset of mRNAs with highly structured 5′ UTR^38^. Other studies have suggested the ability of YTHDC2 to promote TE^33,36^, albeit the underlying mechanism remained elusive. We found that YTHDC2 promotes translation of structured mRNAs by resolving secondary structures. It is possible that YTHDC2 gained this unique feature by possessing motifs that are characteristic of the DEAH/RNA helicase A family. Intriguingly, YTHDC2 has also been shown to accelerate mRNA decay. In particular, YTHDC2 interacts with the 5′ → 3′ exonuclease XRN1 (ref. ^35^). It is conceivable that YTHDC2 relies on CDS methylation signals to coordinate translation and decay of structured mRNAs. Since many of these transcripts encode transcriptional regulators, this coordination offers a means to effectively fine-tune the protein levels in a needed basis. Broadly, CDS methylation forms an important layer of translational regulation by acting as a controllable switch via a specialized m^6^A reader YTHDC2.

## Methods

### Cell lines and reagents

293T and MEF cells were maintained in Dulbecco’s Modified Eagle’s Medium (DMEM) with 10% fetal bovine serum. Antibodies used in this study are listed as follows: anti-YTHDF1 (Abcam ab99080, 1:1,000 WB), anti-YTHDF2 (Proteintech 24744-1-AP, 1:1000 WB), anti-YTHDF3 (Santa Cruz sc-377119, 1:1000 WB), anti-YTHDC2 (Abcam ab176846, 1:1000 WB), anti-METTL3 (Abnova H00056339-B01P, 1:1000 WB), anti-METTL14 (Sigma HPA038002, 1:1000 WB), anti-FTO (Phosphosolutions 597-FTO, 1:1000 WB), anti-ALKBH5 ((Proteintech 16837-1-AP, 1:1000 WB), anti-puromycin (Developmental Studies Hybridoma Bank-PMY-2A4, 1:100 WB), anti-Flag (M2) antibody (F1804), anti-m6A (Millipore ABE572) and anti-β-actin (Sigma-A5441, 1:2,000 WB). [^35^S]-methionine was purchased from PerkinElmer (#NEG772007MC).

### Plasmids

Human YTHDC2 was cloned into pcDNA3 vector. To create the E332Q mutant, site-directed mutagenesis was performed using Q5 Site-Directed Mutagenesis Kit (New England Biolabs) according to the manufacturer manual. Primers: 5′-TGAAGTGCATCAAAGGGATCG-3′ and 5′-CAAGACATCTCGCAATAGG-3′. Mutation was identified by Sanger DNA sequencing.

### Lentiviral shRNAs

shRNA targeting sequences based on RNAi consortium at Broad Institute (http://www.broad.mit.edu/rnai/trc) are listed as below:

YTHDC2 (human): 5′-GCCCACAGATTGGCTTATTTA-3′;

YTHDF1 (human): 5′-CCGCGTCTAGTTGTTCATGAA-3′;

YTHDF2 (human): 5′-CCACAGGCAAGGCCCAATAAT-3′;

YTHDF3 (human): 5′-GATAAGTGGAAGGGCAAATTT-3′;

METTL3 (mouse): 5′-GCTACAGGATGACGGCTTTCT-3′;

METTL14 (mouse): 5′-GGATCAAAGGAA CCGTGAAGC-3′;

FTO (mouse): 5′-GCTGAGGCAGTTCTGGTTTCA-3′;

ALKBH5 (mouse): 5′-GCCTCAGGACATT AAGGAACG-3′;

Scramble control sequence: 5′-AACAGTCGCGTTTGCGACTGG-3′.

shRNA targeting sequences were cloned into DECIPHER^™^ pRSI9-U6-(sh)-UbiC-TagRFP-2A-Puro (Cellecta, CA). Lentiviral particles were packaged using Lenti-X 293T cells (Clontech). Virus-containing supernatants were collected at 48 h after transfection and filtered to eliminate cells. Cells were infected by the lentivirus for 48 h before selection with 1–2 μg ml^−1^ puromycin.

### Immunoblotting

Cells were washed twice with ice-cold phosphate-buffered saline (PBS) and lysed in sodium dodecyl sulfate polyacrylamide gel electrophoresis (SDS-PAGE) sample buffer (50 mM Tris (pH 6.8), 100 mM dithiothreitol, 2% SDS, 0.1% bromophenol blue, 10% glycerol), lysates were heated at 95 °C for 5 min, and then centrifuged at 16,000*g* for 5 min at 4 °C, supernatants were collected and subjected to immunoblotting. Proteins were separated on a SDS-PAGE and transferred to Immobilon-P membranes (Millipore). Membranes were blocked for 1 h in TBS containing 5% nonfat milk and 0.1 % Tween-20, followed by incubation with primary antibodies overnight at 4 °C. After incubation with horseradish peroxidase-coupled secondary antibodies at room temperature for 1 h, immunoblots were visualized using enhanced chemiluminescence (ECLPlus, GE Healthcare).

### Cell proliferation assay

A total of 2000 cells (293T cells with sh-Scramble, sh-YTHDC2, sh-YTHDF1, sh-YTHDF2, sh-YTHDF3, and sh-YTHDC1) per well were seeded into 96-well plates, followed by cell culture for 96 h. The cell viability was detected by adding 10 μl of Cell Counting Kit-8 solution (Dojindo) to each well and the absorbance was detected by TECAN Spak10^TM^ at the wavelength of 450 nm after incubation at 37 °C for 1–2 h.

### Puromycin labeling

Cells at 80–90% confluence were changed with fresh medium 2 h before harvesting, and then were treated with 10 µg ml^−1^ puromycin for 10 min. After washing twice with cold Dulbecco's PBS (DPBS), cells were lysed with SDS-PAGE sample buffer, and proteins were separated on SDS-PAGE and transferred to Immobilon-P membranes. Membranes were blocked for 1 h in TBS containing 5% nonfat milk and 0.1% Tween-20, followed by incubation with puromycin antibodies (1:100 dilution) overnight at 4 °C. After incubation with horseradish peroxidase-conjugated anti-mouse IgG (1:10000 dilution) for 1 h at room temperature, the membrane was visualized using enhanced chemiluminescence.

### [^35^S] Radiolabeling

MEF cells were briefly incubated in methionine- and cysteine-free media before addition of 50 mCi of [^35^S]-methionine. Labeling was stopped by ice-cold DMEM containing 100 mM of cycloheximide. Cells were washed with PBS containing 100 mM of cycloheximide, lysed with polysome lysis buffer. For the quantitation of [^35^S]-Met-labeled proteins, cell lysates were resolved on a 10% Tris-Glycine SDS-PAGE and radiography captured by Typhoon 9400. Quantification of [^35^S] methionine incorporation was done using ImageJ software.

### Dual-luciferase reporter assay

The partial DNA sequence of *Malat1* encoding the typical RNA secondary structure (AACUUAAUGUUUUUGCAUUGGACUUUGAGUU) along with the coding region of Renilla luciferase were cloned into pcDNA-Fluc vector using XbaI and AgeI sites. The stop codon of Fluc and the start codon of Rluc no longer exist. The resulted fusion protein contains an extra 20 amino acids between Fluc and Rluc. The cloning primers were listed as follows: Forward, 5′-GCTCTAGAGTAATTACCAACTTAATGTCCTTGCATTGGACTTTGAGTTATGATTATTTTTTAACTTCGAAAGTT-3′; Reverse, 5′-GCACCGGTTTATTGTTCATTTTTG-3′. The mutant plasmids (A → G and UU → CC) for the pcDNA-Fluc-Malat1SS-Rluc reporter were generated using the Q5® Site-Directed Mutagenesis Kit (New England Biolabs) and primers listed below:

Q5-Mat1SS-(A-G)-F, TTTGCATTGGGCTTTGAGTTATG;

Q5-Mat1SS-(A-G)-R, AACATTAAGTTGGTAATTACTC;

Q5-Mat1SS-(CC-TT)-F, AACTTAATGTTTTTGCATTGGACTTTG;

Q5-Mat1SS-(CC-TT)-R, GGTAATTACTCTAGACACG.

pcDNA-Fluc-Malat1SS-Rluc and its mutant reporters were transfected into control or indicated knockdown MEF cells. After 24 h, Firefly and Renilla luciferase activities in individual cell lysate were measured using Dual-Luciferase® Reporter Assay System (Promega). At least three biological replicates were performed for each cell line and reporter.

### SELECT for detection of m^6^A

Five microgram total RNA was incubated with 40 nM Up Primer, 40 nM Down Primer and 5 μM dNTP in 17 μl 1 × CutSmart buffer (50 mM KAc, 20 mM Tris-HAc, 10 mM MgAc_2_, 100 μg/ml BSA) and annealed in the programs below: 90 °C (1 min), 80 °C (1 min), 70 °C (1 min), 60 °C (1 min), 50 °C (1 min), and 40 °C (6 min). Subsequently, the 17 μl annealing products were incubated with a 3 μl of enzyme mixture containing 0.01 U Bst 2.0 DNA polymerase, 0.5 U SplintR ligase and 10 nmol ATP. The final 20 μl reaction mixture was incubated at 40 °C for 20 min, denatured at 80 °C for 20 min and kept at 4 °C. Quantitative PCR was run at the following condition: 95 °C, 5 min; (95 °C, 10 s; 60 °C, 45 s) × 40 cycles. The SELECT products of indicated site were normalized to the RNA abundance of indicated transcript bearing this site.

### Ribosome profiling

Cells at 80–90% confluence were changed with fresh medium to remove the dead cells 3–4 h before harvesting. Four 10 cm dishes of cells were harvested in 450 µl lysis buffer (10 mM HEPES, pH 7.4, 100 mM KCl, 5 mM MgCl_2_, 1% Triton X-100) containing CHX (100 µg ml^−1^), then centrifuged at 12,000*g*, 4 °C for 10 min. The supernatant was collected and subjected to sucrose gradient sedimentation. Sucrose solutions were prepared in polysome buffer (10 mM HEPES, pH 7.4, 100 mM KCl, 5 mM MgCl_2_). 15–45% (w/v) sucrose density gradients were freshly made in SW41 ultracentrifuge tubes (Backman) using Gradient Master (BioComp Instruments). Totally, 500 µl of the cell lysates was loaded onto sucrose gradients followed by centrifugation for 2.5 h at 32,000 rpm, 4 °C in a SW41 rotor. Separated samples were fractionated at 1.5 ml min^−1^ through an automated fractionation system (Isco) that continually monitors OD254 values.

### RNA-seq and m^6^A-seq

For RNA-seq, 12 µg Trizol extracted total RNAs were fragmentated by using freshly prepared RNA fragmentation buffer (10 mM Tris-HCl, pH 7.0, 10 mM ZnCl_2_) and heating at 94 °C for 5 min, followed by adding 1 µl 0.5 M EDTA to terminate. For m^6^A immunoprecipitation, 1 mg fragmented RNA was incubated with 15 μg anti-m^6^A antibody in 1 × IP buffer (10 mM Tris-HCl, pH 7.4, 150 mM NaCl, and 0.1% Igepal CA-630) for 2 h at 4 °C. The m^6^A-IP mixture was then incubated with Protein A beads for additional 2 h at 4 °C on a rotating wheel. After washing 3 times with IP buffer, bound RNA was eluted using 100 μl elution buffer (6.7 mM *N*^6^-Methyladenosine 5′-monophosphate sodium salt in 1 × IP buffer), followed by ethanol precipitation. Precipitated RNA was used for cDNA library construction and high-throughput sequencing described below.

### cDNA library construction

For Ribo-seq, *E. coli* RNase I (Ambion) was added into the pooled fractions from ribosome profiling (100 U per 100 µl) and incubated at 4 °C for 1 h to convert the polysome into monosome. Total RNAs were extracted using Trizol LS reagent (Invitrogen).

RNase I digested RNA extracts (Ribo-seq) and fragmented RNAs (RNA-seq and m6A-seq) were dephosphorylated for 1 h at 37 °C in a 15 µl reaction (1 × T4 polynucleotide kinase buffer, 10 U SUPERase_In and 20 U T4 polynucleotide kinase). The products were separated on a 15% polyacrylamide TBE-urea gel (Invitrogen) and visualized using SYBR Gold (Invitrogen). Selected regions in the gel corresponding to 40–60 nt (for RNA-seq) or 25–35 nt (for Ribo-seq) were excised. RNA fragments were dissolved by soaking overnight in 400 μl RNA elution buffer (300 mM NaOAc, pH 5.5, 1 mM EDTA, 0.1 U ml^−1^ SUPERase_In). The gel debris was removed using a Spin-X column (Corning), followed by ethanol precipitation. Purified RNA fragments were resuspended in nuclease-free water.

Totally, 0.15 μg linker (rApp/NNNNCTGTAGGCACCATCAAT/3ddC) was added to the RNA fragments, heated at 70 °C for 90 s and then cooled to room temperature, followed by ligation for 3 h at 22 °C in a 20 µl reaction (1 × T4 Rnl2 reaction buffer, 10 U SUPERase_In, 15% PEG8000 and 20 U T4 RNA ligase 2 truncated). The reaction was heat inactivated at 80 °C for 10 min and the products were separated on a 10% polyacrylamide TBE-urea gel and selected regions in the gel corresponding to 65–85 nt (for RNA-seq) or 50–70 nt (for Ribo-seq) were excised. RNA fragments were dissolved by soaking overnight in 400 μl RNA elution buffer, purified RNA fragments were re-suspended in nuclease-free water.

For reverse transcription, the following oligos containing barcodes were used:

(Phos)CTANNNAGATCGGAAGAGCGTCGTGTAGGGAAAGAGTGTAGATCTCGGTGGTCGC(SpC18)CACTCA(SpC18)TTCAGACGTGTGCTCTTCCGATCTATTGATGGTGCCTACAG

(Phos)AGCNNNAGATCGGAAGAGCGTCGTGTAGGGAAAGAGTGTAGATCTCGGTGGTCGC(SpC18)CACTCA(SpC18)TTCAGACGTGTGCTCTTCCGATCTATTGATGGTGCCTACAG

(Phos)ATTNNNAGATCGGAAGAGCGTCGTGTAGGGAAAGAGTGTAGATCTCGGTGGTCGC(SpC18)CACTCA(SpC18)TTCAGACGTGTGCTCTTCCGATCTATTGATGGTGCCTACAG

(Phos)CCGNNNAGATCGGAAGAGCGTCGTGTAGGGAAAGAGTGTAGATCTCGGTGGTCGC(SpC18)CACTCA(SpC18)TTCAGACGTGTGCTCTTCCGATCTATTGATGGTGCCTACAG

where Phos represents phosphorylation, NNN represents random sequence, SpC18 represents Hexa-ethyleneglycol spacer.

The linker ligated RNA sample was mixed with 0.5 mM dNTP and 2.5 mM synthesized primer and incubated at 75 °C for 5 min, followed by incubation on ice for 3 min. The reaction mix was then added with 20 mM Tris (pH 8.4), 50 mM KCl, 5 mM MgCl_2_, 10 mM DTT, 40 U RNaseOUT and 200 U SuperScript III. Reverse transcription reaction was performed according to the manufacturer’s instruction. Reverse transcription products were separated on a 10% polyacrylamide TBE-urea gel. The extended first-strand product band was expected to be approximately 200 nt, and the corresponding region was excised. The cDNA was recovered by using DNA gel elution buffer (300 mM NaCl, 1 mM EDTA). First-strand cDNA was circularized in 20 μl of reaction containing 1 × CircLigase buffer, 2.5 mM MnCl_2_, 1 M Betaine and 100 U CircLigase II (Epicenter). Circularization was performed at 60 °C for 1 h, and the reaction was heat inactivated at 80 °C for 10 min, then was precipitated by ethanol.

### Deep sequencing

Circular template was amplified by PCR by using the Phusion high-fidelity (HF) enzyme (NEB) according to the manufacturer’s instructions. The PCR forward primer: 5′-AATGATACGGCGACCACCGAGATCTACAC-3′ and reverse primer: 5′-CAAGCAGAAGACGGCATACGAGATGTGACTGGAGTTCAGACGTGTGCT CTTCCG-3′

were used to create DNA suitable for sequencing. The PCR contains 1 × HF buffer, 0.2 mM dNTP, 0.5 µM oligonucleotide primers, and 0.5 U Phusion polymerase. PCR was carried out with an initial 30 s denaturation at 98 °C, followed by 12 cycles of 10 s denaturation at 98 °C, 20 s annealing at 65 °C, and 20 s extension at 72 °C. PCR products were separated on a nondenaturing 8% polyacrylamide TBE gel. Expected DNA at 180 bp was excised and recovered. After quantification by Agilent BioAnalyzer DNA 1000 assay, equal amounts of barcoded samples were pooled into one sample. Approximately, 5 pM mixed DNA samples were used for cluster generation followed by sequencing by using sequencing primer 5′-CGACAGGTTCAGAGTTCTACAGTCCGACGATC-3′ (Illumina HiSeq).

### Alignment of sequencing reads

The 3′ adapters and low quality bases were trimmed by Cutadapt^39^. The trimmed reads with length <15 nucleotides were excluded. The remaining reads were mapped to the mouse transcriptome using Bowtie^40^ with parameters: -a --best -m1 --strata. To construct the transcriptome, the annotation file from ENSEMBL database (GRCm38) was used. For each gene, the mRNA with longest CDS was selected. In the case of equal CDS length, the longest transcript was used. For read alignment, a maximum of two mismatches were permitted. To avoid ambiguous, the reads that were mapped to multiple positions were excluded.

### Prediction of m^6^A peak

We used a similar method reported previously to identify m^6^A peaks in the immunoprecipitation sample as compared to the input sample. In brief, a sliding window of 50 nucleotides with a step of 25 nucleotides was employed to scan each transcript. For each window with maximum read coverage higher than 10, a peak-over-median score (POM) was derived by calculating the ratio of the mean read coverage in the window to the median read coverage of corresponding transcript. The windows with POM higher than three in IP sample were obtained. The same processes were performed in input sample. The windows found in input sample were eliminated from following analyses. The windows that overlapped at least single nucleotide were merged into one cluster. Finally, a peak over input (POI) score was assigned to each cluster by calculating the ratio of POM in the IP sample to that in the input sample. The cluster with POI score higher than three were retrieved, and defined as m^6^A-enriched cluster. The peak position with maximum coverage in each m^6^A-enriched cluster was defined as the position of m^6^A peak. The adenosine site of the nearest RRAC motif was defined as m^6^A residue. To reduce noises for background reads and possible bias from peak calling method, only the m^6^A peaks that were found in all biological replicates were used. m^6^A peaks in METTL3 and WTAP knockdown samples were predicted using the same method. The m^6^A peaks with a decrease of POI score up to 50% after METTL3 or WTAP knockdown were defined as WTAP or METTL3 sensitive m^6^A peaks. All m6A sites were classified into different mRNA segments: 5′ UTR, CDS and 3′ UTR, according to the positions of m^6^A. In current study, we mainly focused on CDS m^6^A, therefore m^6^A peaks around the start codon (−15 nt, +100 nt) and the stop codon (−100 nt, +15 nt) were not included in analyses.

### Single nucleotide m^6^A

We used single nucleotide m^6^A to investigate the effect of m^6^A on ribosome movement. The data were obtained from the supplementary file of the study of Linder et al^22^.

### m^6^A-positive mRNAs and negative controls

m^6^A-positive mRNAs refer to the mRNAs that contain at least one m^6^A peak. Nonmethylated mRNAs were defined as the mRNAs that none of the conserved methylation site was found. We found CDS m^6^A tends to be enriched in the transcripts with inactive translation, therefore, to exclude possible biases caused by the difference in basal translational levels, we did not use a full set of non-methylated mRNAs as negative control. Instead, for each m^6^A-positive mRNA, k-nearest neighbors algorithm was used to define a negative control. In brief, we used three non-methylated mRNAs (*K* = 3), which show a similar ribosome density (±10%) to m^6^A positive mRNA in wild type, as negative control. The mean value of nonmethylated mRNAs was used.

When analyzing sequence context and ribosome densities around m^6^A, the nonmethylated adenosine sites of RRAC motifs in the same transcript were defined as negative control. The methylated regions refer to the interval of −400 to +100 nucleotides relative to the methylated codons.

### Ribosome density of transcript

For each transcript, RPKM was used to estimate the ribosome density of transcript. To exclude the effect of RNA level, ribosome density was normalized by corresponding RNA level. mRNAs with RPKM < 1 were excluded.

### Ribosome densities around m^6^A residues

To investigate the effect of m^6^A on ribosome movement, ribosome densities along mRNA regions in the interval of −60 to +60 nucleotides relative to methylated codons were calculated. First, for each methylated codon, footprint reads at each position in the interval of −60 to +60 nucleotides were normalized by the total reads of such region. The regions with total reads <20 were excluded. Second, mean ribosome densities were obtained by averaging the normalized footprint reads at the same positions.

### Identification of regions with ribosomal pausing

To identify structure related ribosomal pausing, we firstly used a sliding window with 30 in length and a step of 3 nt to scan the transcript. Ribosome densities, normalized by the averaged density of CDS, between scramble cells and knockdown cells in the same window were compared. The regions with fold change of knockdown over scramble higher than three were defined as pausing regions. By contrast, the regions with fold change < 1/3 were used as negative controls.

### mRNA secondary structure analysis

For each transcript, a sliding window of 30 nucleotides with a step of 3 nucleotides was used to calculate RNA minimum fold free energy (MFE) along transcript. For each window, MFE was calculated by ViennaRNA^41^, using default parameters. To investigate whether an evolutionally conserved structure is formed in the flanking regions of m^6^A methylation sites, 30 random sequences were generated by shuffling nucleotides of native sliding sequence while controlling for the dinucleotides content^42^. A *z*-score value for each native sequence was calculated as reported before^43^. For aggregation plot, a mean MFE in each window was calculated by averaging MFE values of the windows in the same position. icSHAPE^27^ and PARS^44^, both can capture RNA secondary structure at a transcriptome-wide level, were used to estimate the effect of m^6^A on mRNA folding.

### m^6^A conservation analysis

For m^6^A between HEK293 and MEF, we retrieved orthologous sequences from ENSEMBL, only one-to-one orthologs were used. The orthologs were then aligned by Muscle^45^ using default parameters. The positions of m^6^A peaks were adjusted according to the positions of insertions and deletions between orthologs. The adjusted m^6^A peaks that were found at the same position of the aligns were defined as conserved m^6^A. m^6^A peaks only found in human or mouse were defined non-conserved m^6^A.

### Analysis of PAR-CLIP

After filtering out the low-quality reads, the remaining PAR-CLIP reads were aligned to human genome using bowtie, with parameters: −v 2 −m 10 --best --strata. PAR-CLIP peaks were predicted by PARalyzer using default parameters. The genome coordinates of PAR-CLIP peaks were mapped to coordinates of the longest transcript using custom Perl script.

### Motif analysis

Motif analyses were performed by MEME^46^.

### Gene ontology (GO) analysis

GO analyses were performed by DAVID GO.

### tAI calculation

The tRNA adaptation index (tAI) was calculated using the method reported before^47^. tRNA copy number used for tAI calculation was downloaded from the GtRNAdb database.

### Reporting summary

Further information on research design is available in the Nature Research Reporting Summary linked to this article.

## Supplementary information

Supplementary Information

Peer Review

Reporting summary

Description of Additional Supplementary Files

Supplementary Data 1

## Data Availability

All new sequencing data that support the findings of this study have been deposited in the National Center for Biotechnology Information Gene Expression Omnibus (GEO) and are accessible through the GEO Series accession number GSE129194. All other published sequencing data have been cited in main text, the GEO Series accession numbers of published sequencing data are listed in Supplementary Data 1. All other relevant data are available from the corresponding author on request. The source data underlying Fig. 4a and Supplementary Fig. 9a are provided as Source Data files.

## Source Data

Source Data

## Code availability

All the procedures but sequencing mapping were completed using custom Perl scripts, which are available upon request.
