# Supplementary material for: m6A in mRNA coding regions promotes translation via the RNA helicase-containing YTHDC2
Source: Nat Commun. 2019 Nov 25;10:5332. doi: 10.1038/s41467-019-13317-9 (PMC6877647; doi:10.1038/s41467-019-13317-9)
Supplement: Supplementary file 1 — Supplementary Information [file 41467_2019_13317_MOESM1_ESM.pdf]

Supplementary Information

m^6A in mRNA coding regions promotes translation via the RNA helicase-containing YTHDC2

Mao et al.

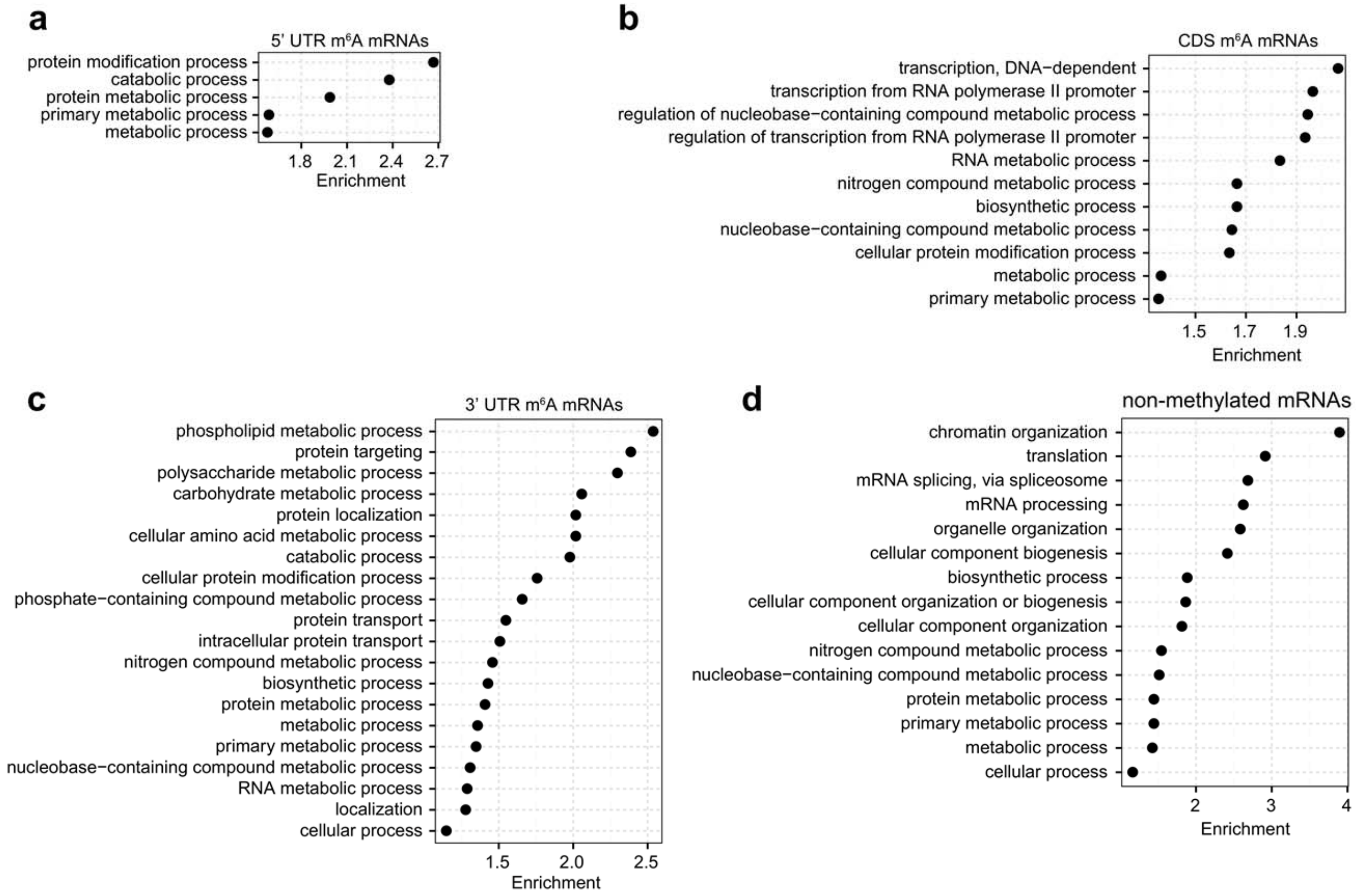

Supplementary Figure 1

Gene Ontology (GO) analysis for transcripts with regional methylation.

Transcripts are stratified based on regional methylation in the form of m^6A (**a**, 5'UTR; **b**, CDS, **c**, 3'UTR, **d**, no-methylation). GO analysis is conducted for each group using biological process enrichment. Only the significantly enriched GO terms (FDR < 0.01) are shown.

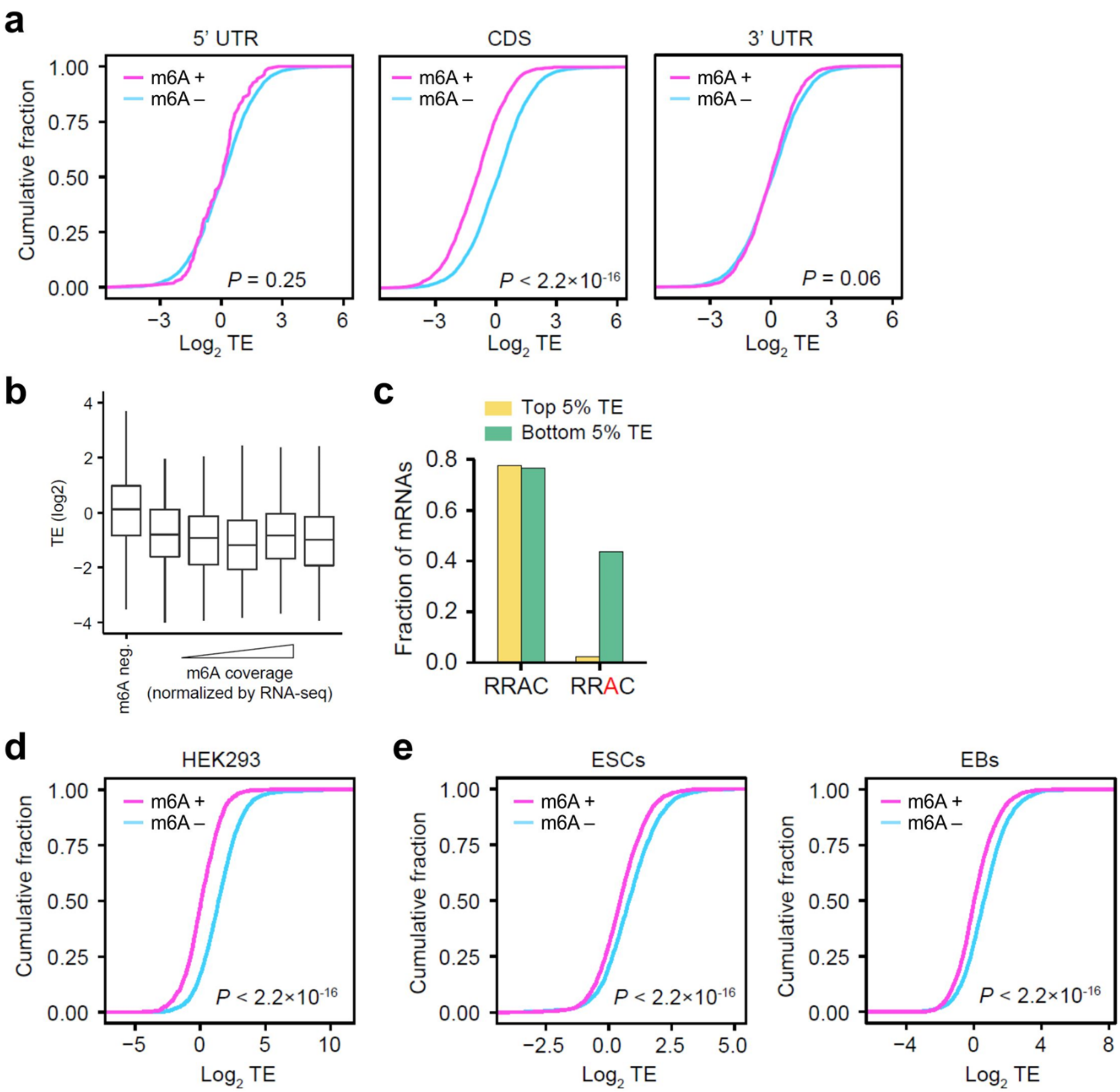

Supplementary Figure 2

Characterization of regional m^{6}A modification.

a. Transcripts are stratified based on regional methylation in the form of m^{6}A (5' UTR, CDS and 3' UTR). Translation efficiency (TE) is plotted as accumulative fractions for transcripts with regional (m^{6}A +, pink line) and without (m^{6}A –, blue line) m^{6}A modification. Ribo-seq and RNA-seq data from MEF cells are used. Wilcox test is performed for statistical tests in **a**, **d** and **e**.

b. A box plot shows the relative translation efficiency for transcripts with different m^{6}A coverage normalized by RNA abundance.

c. Transcripts with top and bottom 5% TE were selected followed by grouping based on CDS methylation. mRNAs with at least one m6A consensus motif in CDS or CDS m6A peak were counted, respectively. The m^{6}A site is highlighted by red.

d – e. Translation efficiency (TE) is plotted as accumulative fractions for mRNAs with CDS m^{6}A modification (m^{6}A +, pink line) and non-methylated mRNAs (m^{6}A –, blue line). **(d)** shows TE in HeLa cells. **(e)** shows TE in ESCs (left) and EBs (right) cells. Source data are provided as a Source Data file.

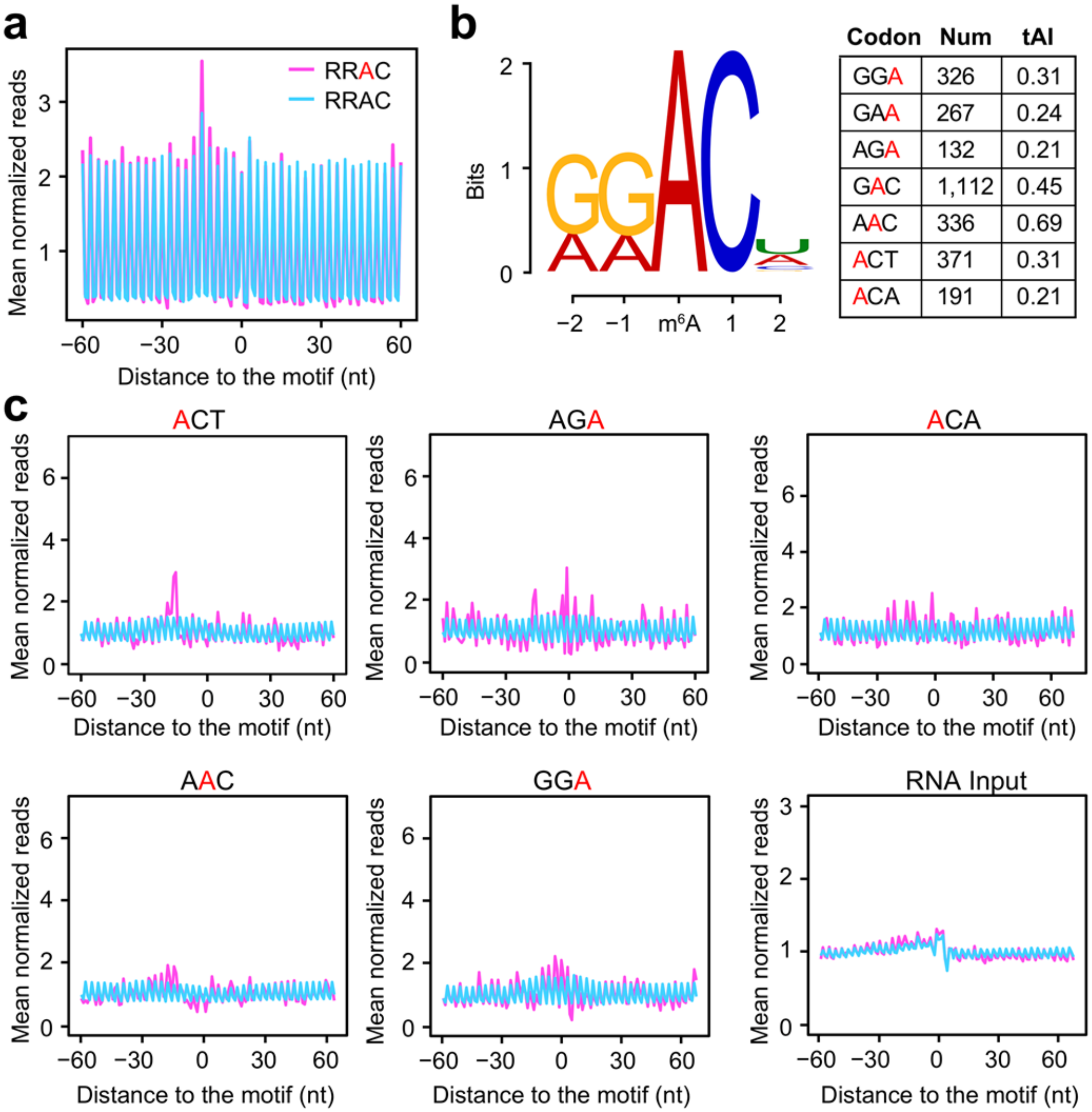

Supplementary Figure 3

Characterization of ribosomal pausing on m^{6}A methylated codons.

a. Aggregation plots show the mean ribosome densities along mRNA regions aligned to the RRAC motif with (pink line) or without (blue line) m^{6}A modification. Ribo-seq data from MEF cells are used. m^{6}A sites are predicted using m^{6}A-seq data in MEF cells. The methylated sites are labeled as red.

b. Left panel shows m^{6}A consensus motif identified by miCLIP-m6A-seq in HEK239 cells. The right table shows frequencies and codon optimal index, measured by tAI, of codons with m^{6}A modification at different positions of codon. The methylated sites are labeled as red.

c. Aggregation plots show the mean ribosome densities and RNA input (right bottom) along mRNA regions aligned to the codons with (pink line) or without (blue line) m^{6}A modification. The methylated sites are labeled as red. Source data are provided as a Source Data file.

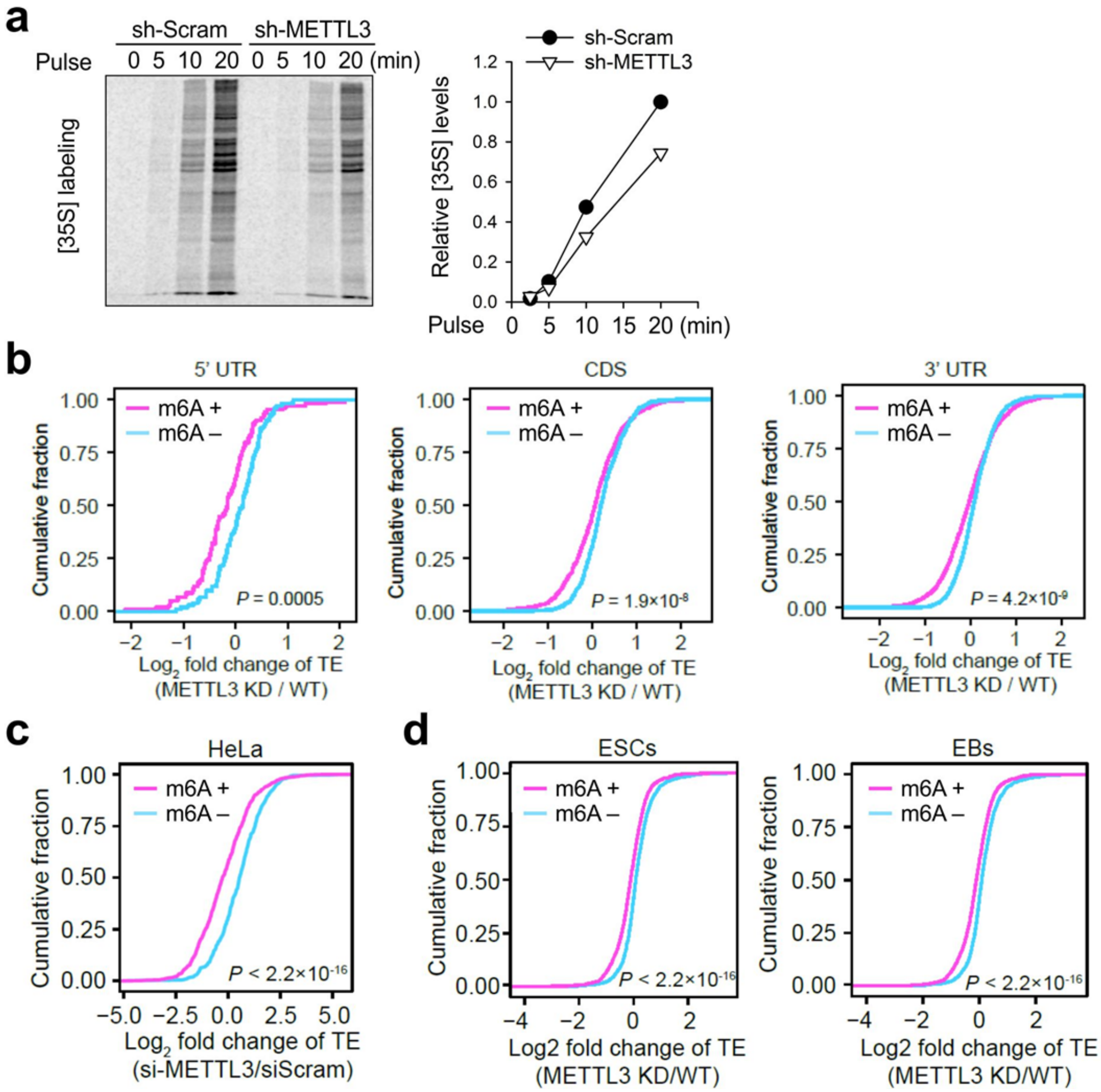

Supplementary Figure 4

Potential roles of METTL3 in translation efficiency of transcripts with regional m^{6}A modification.

a. Global protein synthesis in MEF cells with or without METTL3 knockdown is measured by [35S] autoradiograph. The right panel shows quantification of [35S] autoradiograph with or without METTL3 knockdown.

b. Transcripts are stratified based on regional methylation in the form of m^{6}A in MEF cells (5' UTR, CDS and 3' UTR). The fold change of translation efficiency upon METTL3 knockdown is plotted as accumulative fractions for mRNAs with regional (m^{6}A +, pink line) or without m^{6}A modification (m^{6}A –, blue line). Both groups have similar levels of basal TE. Wilcox test is performed for statistical tests in **b**, **c**, and **d**.

c. The fold change of translation efficiency upon METTL3 knockdown is plotted as accumulative fractions for mRNAs bearing CDS methylation (m^{6}A +, pink line) or not (m^{6}A –, blue line). Ribo-seq and m^{6}A-seq data from HeLa cells are used. To exclude side-effect of m^{6}A reader YTHDF1 or YTHDF3, which also promotes translation efficiency, transcripts targeted by YTHDF1 or YTHDF3 are not included in m^{6}A +.

d. The fold change of translation efficiency upon METTL3 knockdown is plotted as accumulative fractions for mRNAs bearing CDS methylation (m^{6}A +, pink line) or not (m^{6}A –, blue line) in ESCs (left panel) and EBs (right panel) cells. Source data are provided as a Source Data file.

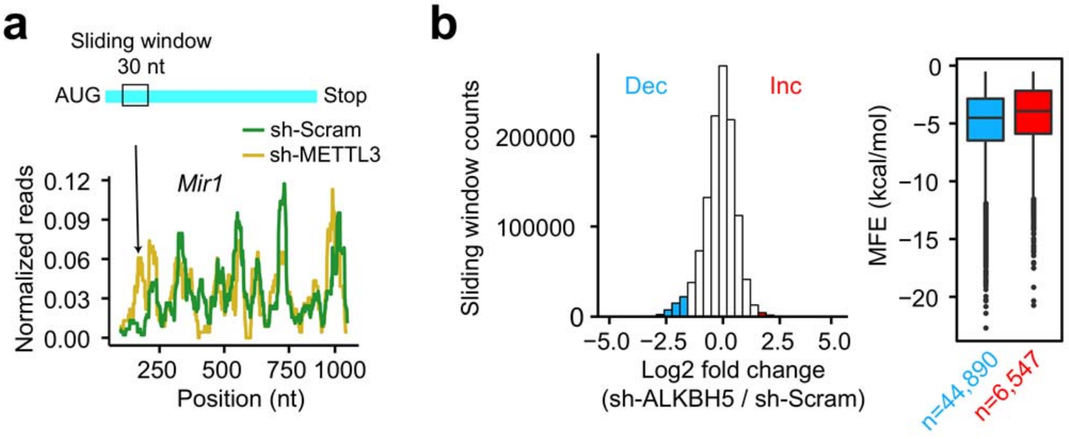

Supplementary Figure 5

Roles of ALKBH5 in ribosomal pausing on transcripts with regional m^6A methylation.

a. Schematic diagram of identification on ribosomal pausing regions. A sliding window of 30 nt in length with a step of 3 nt is used to calculate the local ribosome density. Regions with >3 fold (Inc) changes are defined as ribosomal pausing regions. Regions with <1/3 fold (Dec) changes are also extracted, and used as negative control. Data from the transcript *Mir1* in MEF cells with or without METTL3 knockdown are shown.

b. A histogram shows the distribution of changes of regional ribosome density in response to ALKBH5 knockdown. Regions with <1/3 fold (Dec, blue) and >3 fold (Inc, red) changes are highlighted by color coding. The right box plot shows the predicted minimum folding free energy (MFE) for regions with <1/3 fold (Dec) and >3 fold (Inc) changes (Wilcox test, $P < 2.2\times10^{-16}$). The median of MFE in each group is indicated by a center line, the box shows the upper and lower quantiles, whiskers shows the 1.5x interquartile range, and the outliers are indicated by points. Source data are provided as a Source Data file.

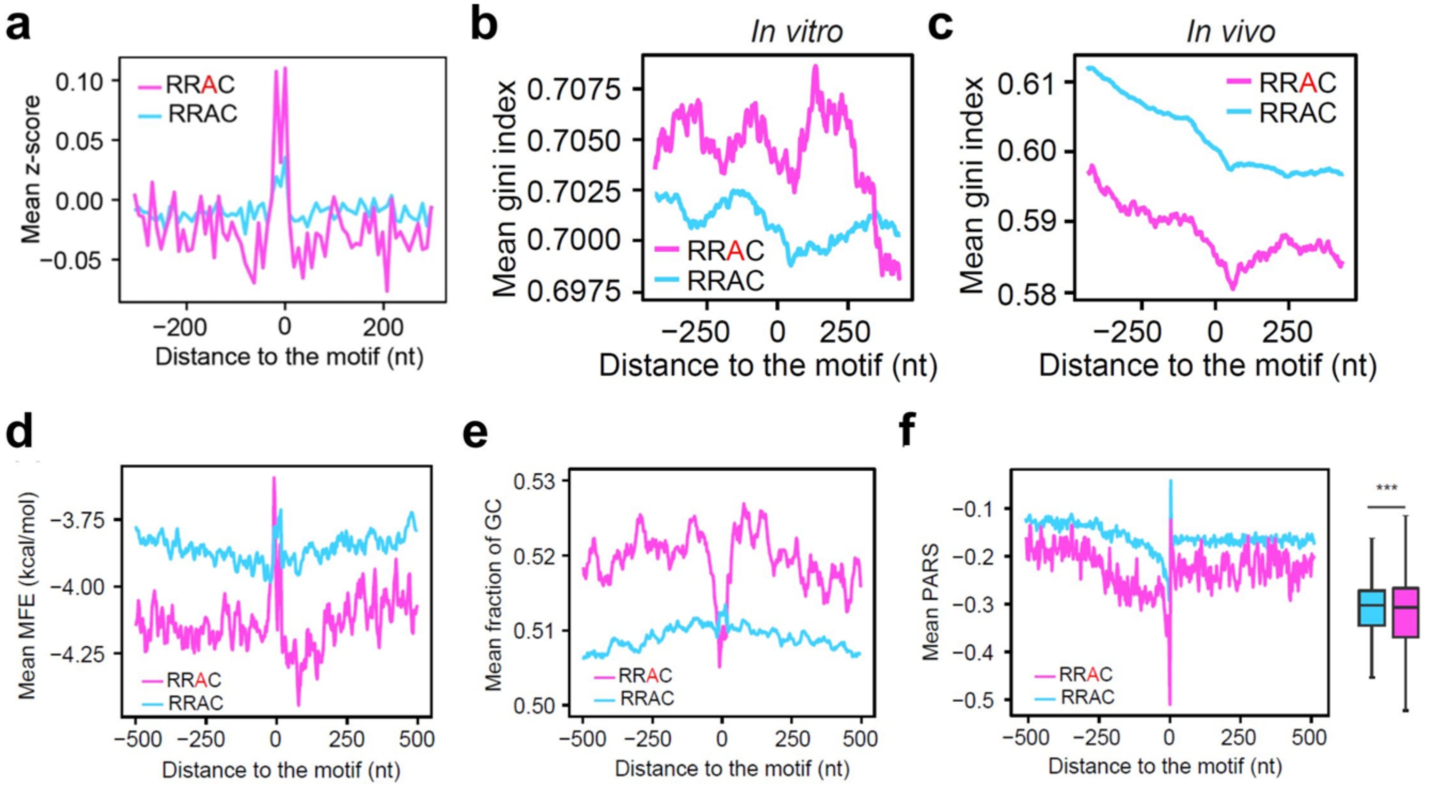

Supplementary Figure 6

Structural characterization of transcripts with regional m^{6}A methylation.

a. The z-score of mean folding energy (MFE) around the m6A sites was calculated by using a sliding window with 30 nt in length and a step of 3nt. For each sequence, 30 random sequences were generated by shuffling nucleotides while keeping dinucleotide content. The methylated sites in **a - f** are labeled as red.

b. The Gini index of *in vitro* icSHAPE signals is plotted along mRNA regions surrounding the RRAC motif with (pink) or without (blue) m^{6}A modification.

c. The Gini index of *in vivo* icSHAPE signals around methylated and non-methylated regions. Notably, a low *Gini index* indicates a less structured region.

d. The predicted minimum folding free energy (MFE) is plotted along mRNA regions surrounding the RRAC motif with (pink line) or without (blue line) m^{6}A modification. Notably, a lower MFE value indicates a higher potential for RNA secondary structures.

e. The GC content is plotted along mRNA regions surrounding the RRAC motif with (pink line) or without (blue line) m^{6}A modification. Notably, the RRAC motif shows the comparable GC content due to the same consensus sequence.

f. The *in vitro* PARS signal is plotted along mRNA regions surrounding the RRAC motif with (pink) or without (blue) m^{6}A modification. Notably, a lower *in vitro* PARS signal indicates a less structured region. The median of PARS scores in each group is indicated by a center line, the box shows the upper and lower quantiles, whiskers shows the 1.5x interquartile range. The outliers are not shown. *** Wilcox test, $P < 0.001$. Source data are provided as a Source Data file.

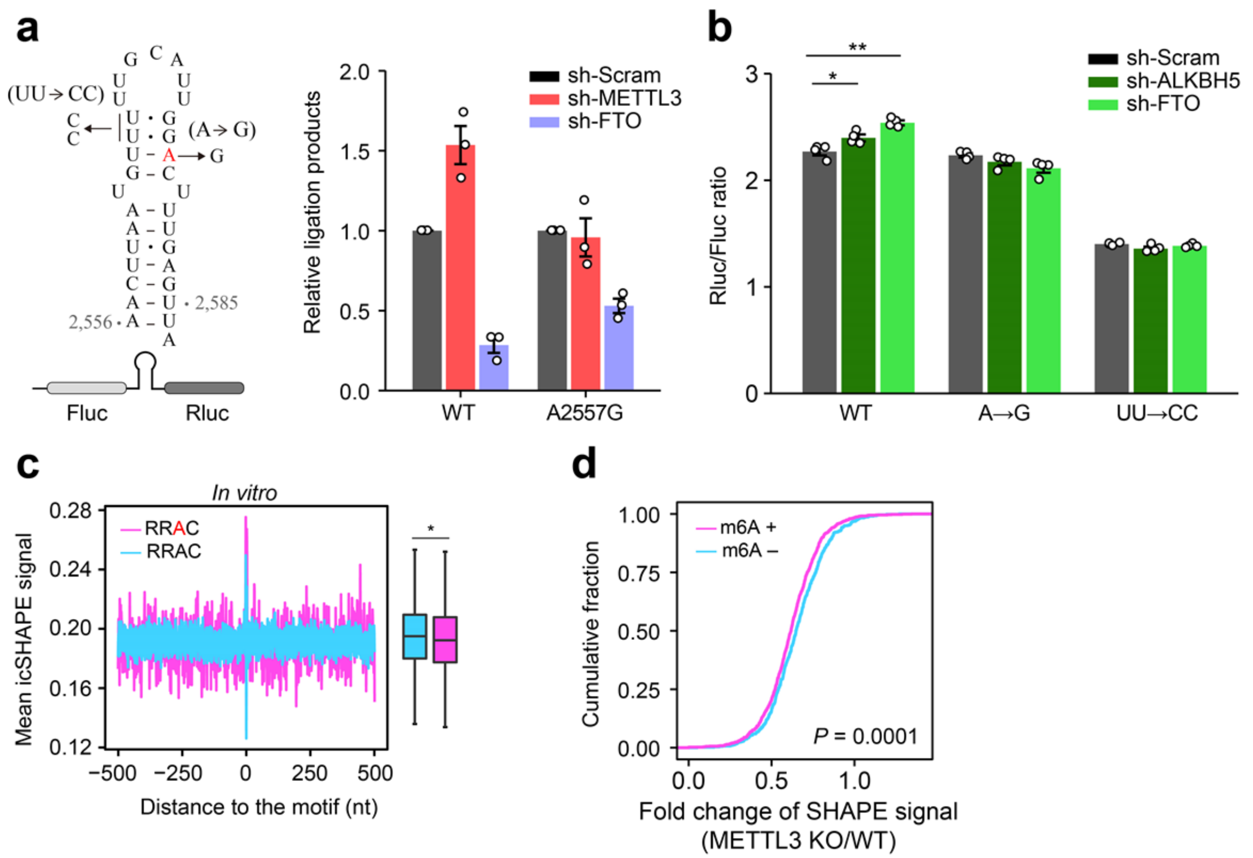

Supplementary Figure 7

Translational evaluation of mRNAs with m^{6}A-influenced secondary structures.

a. The left panel shows the schematic of a dual luciferase reporter with a sandwiched secondary structure derived from *MALAT1* (2,556 – 2,587). Both UU→CC and A→G mutants are also shown. The m^{6}A modification site is labeled as red. The right panel shows the measurement of m6A levels on A2577 of Malat1 in MEF cells transfected with luciferase reporters with WT or A2577G mutation. Notably, lowered ligation products indicate higher m^{6}A levels. Data are presented as mean ± s.e.m. from three replicates. *, $P < 0.05$. The observed values for each biological replicates are shown as points in **a** and **b**.

b. The ratio of Rluc/Fluc in transfected cells expressing wild type or indicated mutants, with either FTO or ALKBH5 knockdown. Standard deviations are shown as error bars. Single-tailed *t*-test, * $P < 0.05$, ** $P < 0.01$.

c. The *in vitro* icSHAPE signal is plotted along mRNA regions surrounding the RRAC motif with (pink) or without (blue) m^{6}A modification. Notably, a higher *in vitro* icSHAPE signal indicates a less structured region. The right boxplot shows the average of icSHAPE signals across mRNA regions from -500 nt to 500 nt relative to the RRAC motif with (pink) or without (blue) m^{6}A modification. The median of icSHAPE signals in each group is indicated by a center line, the box shows the upper and lower quantiles, whiskers shows the 1.5x interquartile range. The outliers are not shown. * Wilcox test, $P < 0.05$. The methylated sites in a-f are labeled as red.

d. Using the icSHAPE data sets derived from mESC cells, the fold change of icSHAPE signals upon METTL3 knockout is plotted as accumulative fractions for mRNAs bearing CDS methylation (m6A +) or not (m6A –) (Wilcox test, P = 0.0001). Source data are provided as a Source Data file.

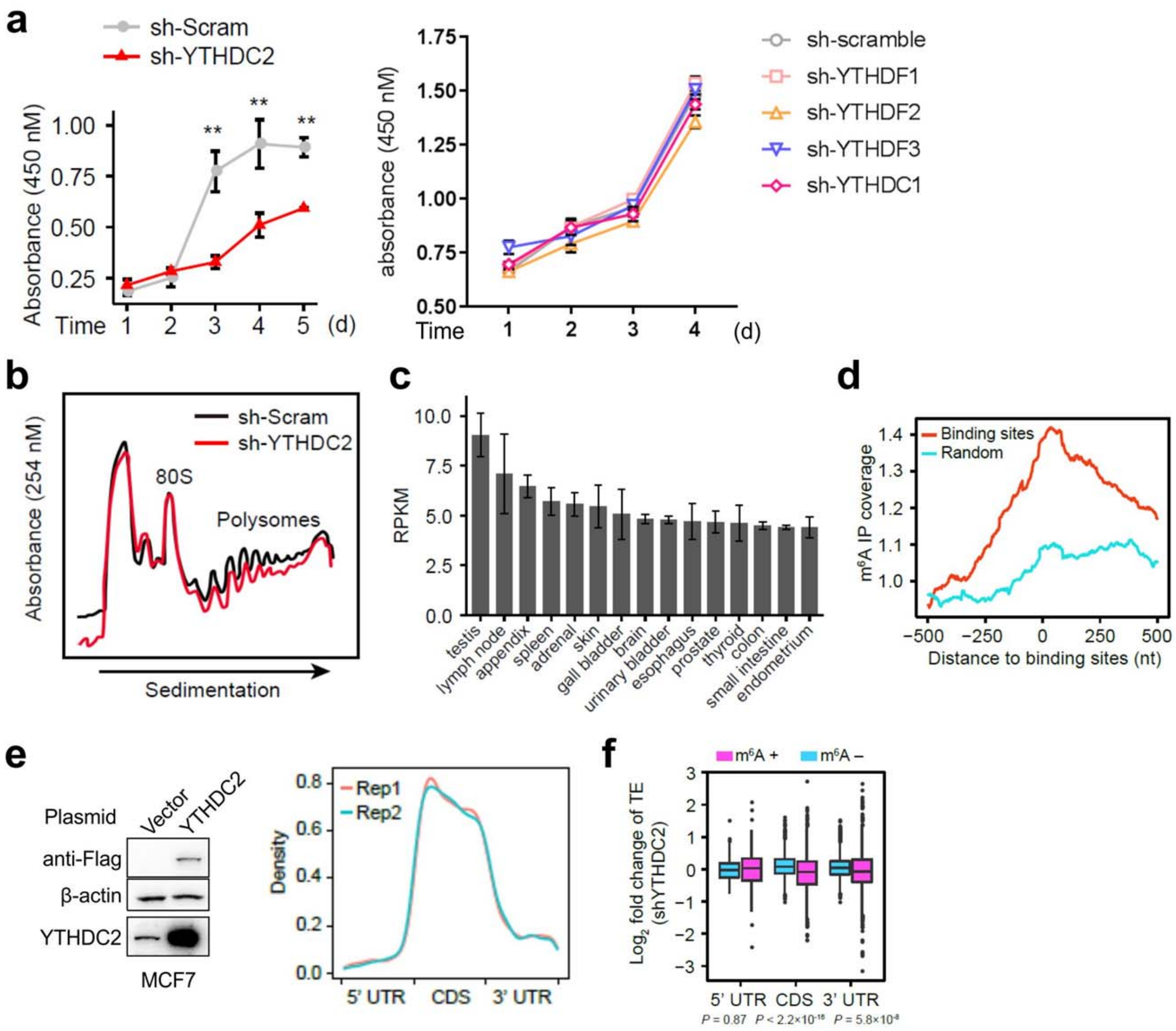

Supplementary Figure 8

Characterization of YTHDC2 in mRNA translation.

a. Cell growth rate was measured in HEK293 cells lacking each individual m^{6}A reader proteins. Notably, only YTHDC2 knockdown significantly reduced the growth rate. *t*-test, ** *P* < 0.01. Error bars, mean ± s.e.m.

b. Polysome profiles of HEK293 cells before and after YTHDC2 knockdown.

c. Expression levels of YTHDC2, measured by RPKM of RNA-seq, in different tissues. Standard deviations are shown as error bars.

d. Aggregation plot shows the mean m^{6}A IP coverage along mRNA regions aligned to the YTHDC2 binding sites (red line) or the sites randomly picked up from the transcript (Random, blue line) in HeLa cells.

e. Distribution of the binding sites of YTHDC2 across the human transcriptome. All binding sites are identified from PAR-CLIP data sets obtained from MCF7 cells transfected with Flag-tagged YTHDC2 (left panel).

f. Transcripts are stratified based on regional methylation in the form of m^6A in MEF cells (5’ UTR, CDS and 3’ UTR). The fold change of translation efficiency upon YTHDC2 knockdown is plotted as accumulative fractions for mRNAs with regional (m^6A +, pink line) or without m^6A modification (m^6A –, blue line). Notably, only mRNAs bearing methylated CDS shows dramatically reduction of translation upon YTHDC2 depletion. YTHDC2 knockdown has little effect on the translation of mRNAs with 5’ UTR or 3’UTR methylation. The median of fold change values in each group is indicated by a center line, the box shows the upper and lower quantiles, whiskers shows the 1.5x interquartile range. The outliers are not shown. Wilcox test is performed for statistical tests. Source data are provided as a Source Data file.

8

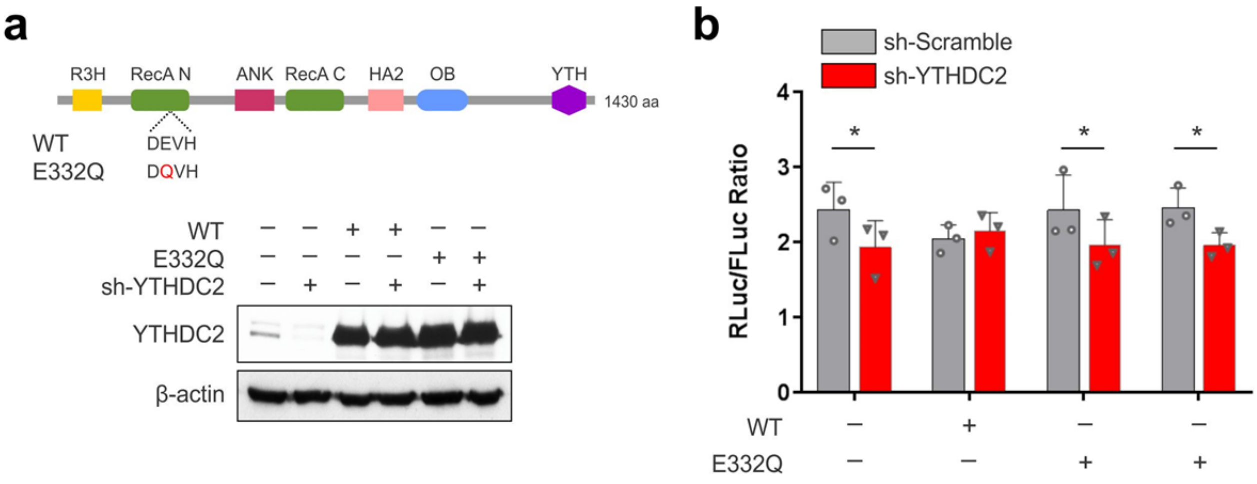

Supplementary Figure 9

Rescuing effects of YTHDC2 in mRNA translation.

a. Schematic view of human YTHDC2 domain structures with the helicase domain RecA highlighted in wild type (WT) and E332Q mutant. The bottom panel shows the expression levels of exogenous human YTHDC2 (WT and E332Q) in HEK293 cells with YTHDC2 knockdown.

b. HEK293 cells with or without YTHDC2 knockdown were added back WT YTHDC2 or the E332Q mutant together with the plasmid expressing the dual luciferase reporter. The Rluc/Fluc ratio is shown after normalization to the Scramble control. Error bars, mean ± s.e.m.; Single-tailed t-test, * $P < 0.05$.

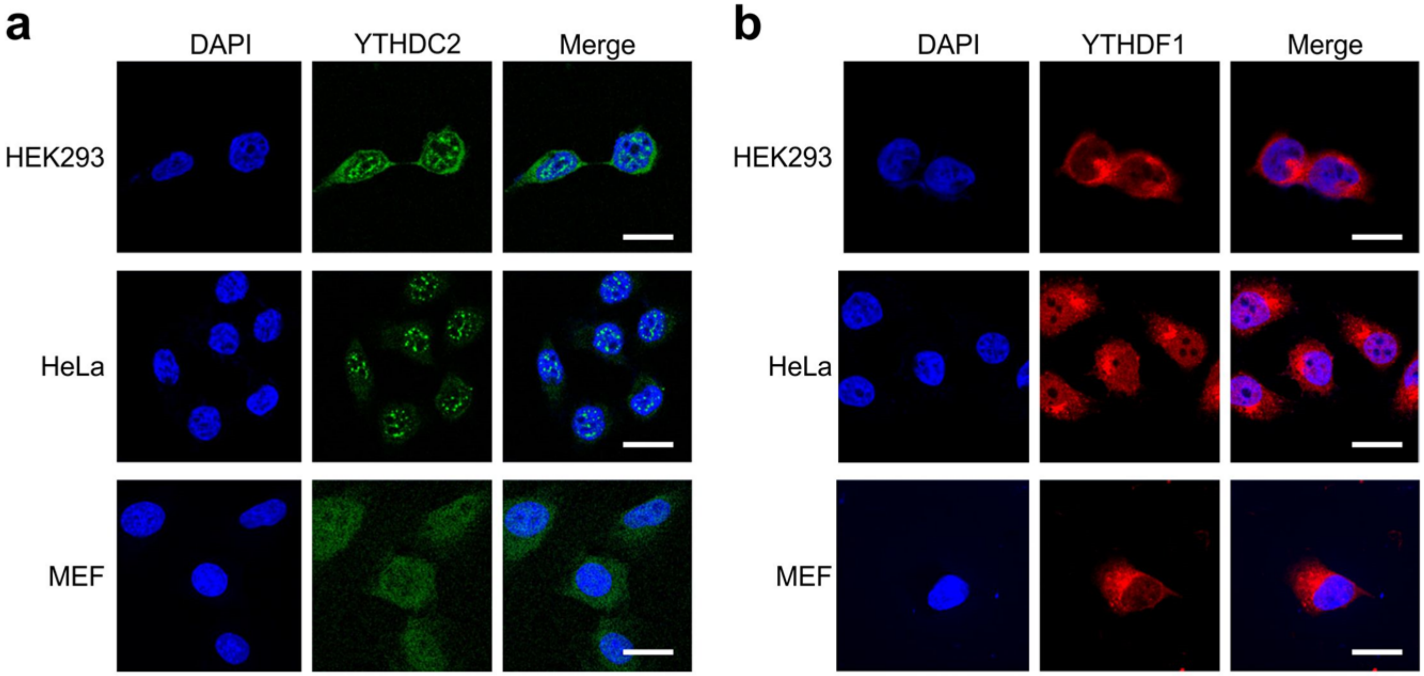

Supplementary Figure 10

Subcellular localization of YTHDC2 and YTHDF1 in different cell lines.

Endogenous YTHDC2 **(a)** and YTHDF1 **(b)** in HEK293, HeLa, and MEF cells were Immuno-stained using antibodies. Nuclei was counter-stained with DAPI.
