# Supplementary material for: m6A in mRNA coding regions promotes translation via the RNA helicase-containing YTHDC2
Source: Nat Commun. 2019 Nov 25;10:5332. doi: 10.1038/s41467-019-13317-9 (PMC6877647; doi:10.1038/s41467-019-13317-9)
Supplement: Supplementary file 3 — Reporting summary [file 41467_2019_13317_MOESM3_ESM.pdf]

nature research

Corresponding author(s): Shu-Bing Qian
NCOMMS-19-09738B

Last updated by author(s): Oct 11, 2019

# Reporting Summary

Nature Research wishes to improve the reproducibility of the work that we publish. This form provides structure for consistency and transparency in reporting. For further information on Nature Research policies, see Authors & Referees and the Editorial Policy Checklist .

## Statistics

For all statistical analyses, confirm that the following items are present in the figure legend, table legend, main text, or Methods section.

| n/a | Confirmed | |
| --- | --- | --- |
| ☐ | ☒ | The exact sample size (*n*) for each experimental group/condition, given as a discrete number and unit of measurement |
| ☐ | ☒ | A statement on whether measurements were taken from distinct samples or whether the same sample was measured repeatedly |
| ☐ | ☒ | The statistical test(s) used AND whether they are one- or two-sided<br>*Only common tests should be described solely by name; describe more complex techniques in the Methods section.* |
| ☐ | ☒ | A description of all covariates tested |
| ☐ | ☐ | A description of any assumptions or corrections, such as tests of normality and adjustment for multiple comparisons |
| ☐ | ☒ | A full description of the statistical parameters including central tendency (e.g. means) or other basic estimates (e.g. regression coefficient) AND variation (e.g. standard deviation) or associated estimates of uncertainty (e.g. confidence intervals) |
| ☐ | ☒ | For null hypothesis testing, the test statistic (e.g. *F*, *t*, *r*) with confidence intervals, effect sizes, degrees of freedom and *P* value noted<br>*Give P values as exact values whenever suitable.* |
| ☒ | ☐ | For Bayesian analysis, information on the choice of priors and Markov chain Monte Carlo settings |
| ☒ | ☐ | For hierarchical and complex designs, identification of the appropriate level for tests and full reporting of outcomes |
| ☐ | ☒ | Estimates of effect sizes (e.g. Cohen's *d*, Pearson's *r*), indicating how they were calculated |

*Our web collection on statistics for biologists contains articles on many of the points above.*

## Software and code

Policy information about availability of computer code

| | |
| --- | --- |
| Data collection | Polysome profiling data were collected by automated fractionation system Isco that continually monitors OD254 values. [35S} Radio-labeling data were collected by ImageJ software. Firefly and Renilla luciferase activities were collected by Dual-Luciferase Reporter Assay System (Promega). Immunoblots were visualized using enhanced chemiluminescence (ECL plus, GE Healthcare). High through-put sequencing data was collected by illumina HiSeq Control Software v2.2.58 for HiSeq2500 System. |
| Data analysis | Cutadapt v1.18 was used to filter low quality reads. Bowtie v1.1.2 was used to align reads to human and mouse transcriptome. R v3.5.1 was used to perform all statistical analysis. ggplot2 for R was used to make all statistical figures. RNAfold v2.1.9 was used to predict RNA structures. Muscle v3.8.31 was used to align othologs for evolutionary analysis. PARalyzer v1.5 was used to analyze PAR-CLIP data. MEME v4.11.2 was used to motif analysis. The custom Perl scripts was used to analyze Ribo-seq, RNA-seq and m6A-seq data, which are available on request to the corresponding authors. |

For manuscripts utilizing custom algorithms or software that are central to the research but not yet described in published literature, software must be made available to editors/reviewers. We strongly encourage code deposition in a community repository (e.g. GitHub). See the Nature Research guidelines for submitting code & software for further information.



October 2018

## Data

Policy information about availability of data

All manuscripts must include a data availability statement. This statement should provide the following information, where applicable:

- Accession codes, unique identifiers, or web links for publicly available datasets
- A list of figures that have associated raw data
- A description of any restrictions on data availability

All new sequencing data that support the findings of this study have been deposited in the National Center for Biotechnology Information Gene Expression Omnibus (GEO) and are accessible through the GEO Series accession number GSE129194. All other published sequencing data have been cited in main text, the GEO Series accession numbers of published sequencing data are listed in Supplementary Table 1. All other relevant data are available from the corresponding author on request.

# Field-specific reporting

Please select the one below that is the best fit for your research. If you are not sure, read the appropriate sections before making your selection.

[x] Life sciences [ ] Behavioural & social sciences [ ] Ecological, evolutionary & environmental sciences

For a reference copy of the document with all sections, see nature.com/documents/nr-reporting-summary-flat.pdf

# Life sciences study design

All studies must disclose on these points even when the disclosure is negative.

| | |
|---|---|
| Sample size | No sample size was per-determined. Three or more independent results were used to perform statistical analysis. Two or more biological replicates for sequencing Ribo-seq and RNA-seq were performed. All sample sizes and the number of replicates were stated in figure legends. |
| Data exclusions | No data were excluded from analysis. |
| Replication | Experiments in this study were reproduced at least two times. Replication were described in figure legends. |
| Randomization | No randomization was used. |
| Blinding | Investigators were blinded to group allocation during data collection and analysis. |

# Reporting for specific materials, systems and methods

We require information from authors about some types of materials, experimental systems and methods used in many studies. Here, indicate whether each material, system or method listed is relevant to your study. If you are not sure if a list item applies to your research, read the appropriate section before selecting a response.

## Materials & experimental systems

| n/a | Involved in the study | |
|---|---|---|
| [ ] | [x] | Antibodies |
| [ ] | [x] | Eukaryotic cell lines |
| [x] | [ ] | Palaeontology |
| [x] | [ ] | Animals and other organisms |
| [x] | [ ] | Human research participants |
| [x] | [ ] | Clinical data |

## Methods

| n/a | Involved in the study | |
|---|---|---|
| [x] | [ ] | ChIP-seq |
| [x] | [ ] | Flow cytometry |
| [x] | [ ] | MRI-based neuroimaging |

## Antibodies

| | |
|---|---|
| Antibodies used | anti-YTHDF1 (Abcam ab99080, 1:1,000 WB), anti-YTHDF2 (Proteintech 24744-1-AP, 1:1,000 WB), anti-YTHDF3 (Santa Cruz sc-377119, 1:1,000 WB), anti-YTHDC2 (Abcam ab176846, 1:1,000 WB), anti-METTL3 (Abnova H00056339-B01P, 1:1000 WB), anti-METTL14 (Sigma HPA038002, 1:1000 WB), anti-FTO (Phosphosolutions 597-FTO, 1:1000 WB), anti-ALKBH5 ((Proteintech 16837-1-AP, 1:1000 WB), anti-puromycin (Developmental Studies Hybridoma Bank-PMY-2A4, 1:100 WB), anti-Flag (M2) antibody (F1804), anti-m6A (Millipore ABE572) and anti-β-actin (Sigma-A5441, 1:2,000 WB). |
| Validation | Antibodies were validated as noted on manufacturer's website or as cited in the results/Methods sections. |

## Eukaryotic cell lines

Policy information about cell lines

| | |
|---|---|
| Cell line source(s) | All cell lines were purchased from the American Type Culture Collection (ATCC). |
| Authentication | Cell lines were not authenticated by ourselves. |
| Mycoplasma contamination | All cell lines were tested to be mycoplasma negative. |
| Commonly misidentified lines (See ICLAC register) | No cell lines used in this study were found in the database of commonly misidentified cell lines that is maintained by ICLAC and NCBI Biosample. |
