# Supplementary material for: m6A in mRNA coding regions promotes translation via the RNA helicase-containing YTHDC2
Source: Nat Commun. 2019 Nov 25;10:5332. doi: 10.1038/s41467-019-13317-9 (PMC6877647; doi:10.1038/s41467-019-13317-9)
Supplement: Supplementary file 4 — Description of Additional Supplementary Files [file 41467_2019_13317_MOESM4_ESM.docx]

**Description of Additional Supplementary Files**

File Name: Supplementary Data 1

Description: lists all published sequencing data cited in the manuscript, together with the GEO accession numbers
