# Supplementary figures and images for: m6A in mRNA coding regions promotes translation via the RNA helicase-containing YTHDC2
Source: Nat Commun. 2019 Nov 25;10:5332. doi: 10.1038/s41467-019-13317-9 (PMC6877647; doi:10.1038/s41467-019-13317-9)

Fig 4a

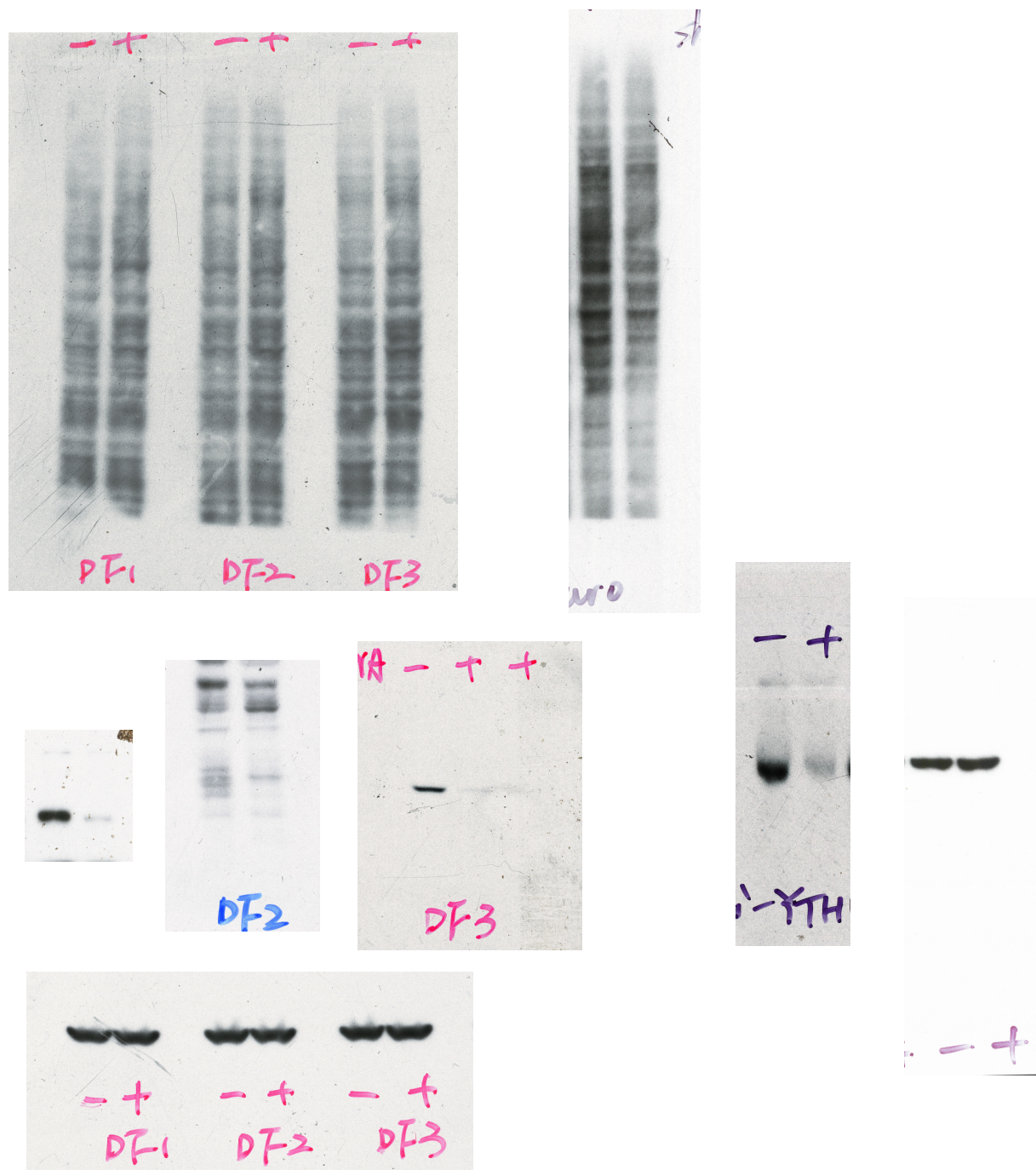

Fig S9a

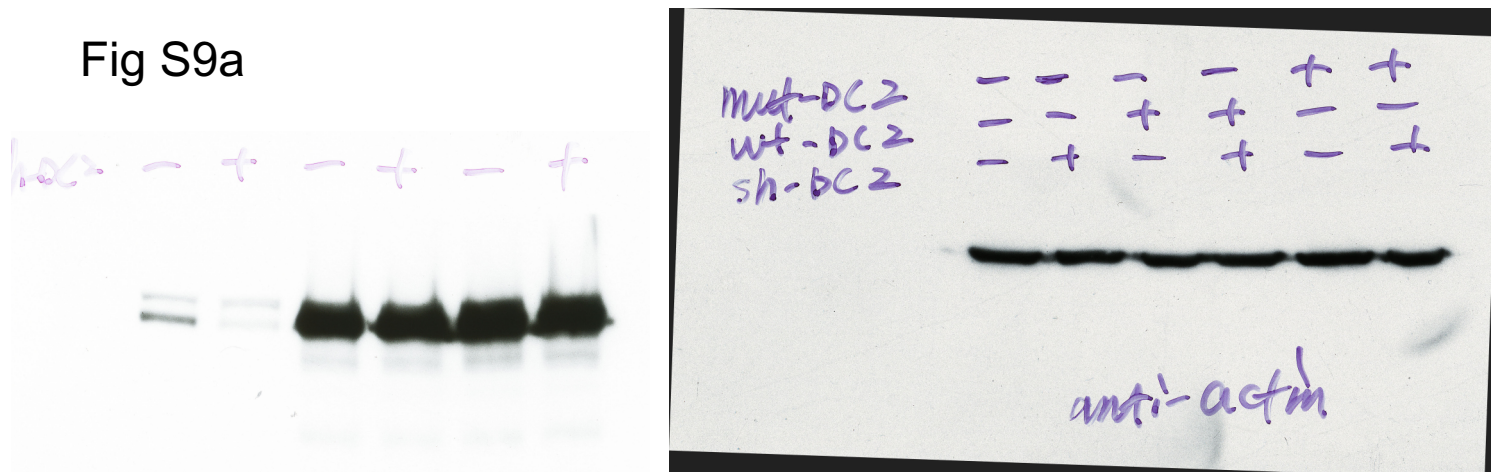

Supplement: Supplementary file 6 — Source Data [file 41467_2019_13317_MOESM6_ESM.zip › Source data 1.pdf]
