# Supplementary material for: m6A in mRNA coding regions promotes translation via the RNA helicase-containing YTHDC2
Source: Nat Commun. 2019 Nov 25;10:5332. doi: 10.1038/s41467-019-13317-9 (PMC6877647; doi:10.1038/s41467-019-13317-9)
Supplement: Supplementary file 2 — Peer Review [file 41467_2019_13317_MOESM2_ESM.pdf]

Reviewers' comments:

Reviewer #1 (Remarks to the Author):

In this manuscript, Mao et. al. investigated how m6A methylation at mRNA coding regions (CDS) modulates ribosome dynamics and translation. The authors reported that CDS m6A facilitates translation by opening up mRNA secondary structures, and suggested this process is mediated through the m6A reader YTHDC2 which contains RNA helicase domains.

This work contains new information very useful to the community. It addresses the regional effects of m6A and helps clarify the role of m6A on translational regulation. It also represents a good example of "correlation does not imply causation"—something many should keep in mind when interpreting functional significance of nucleic acid modifications from high-throughput sequencing data. I am in favor of publishing this work if the following comments can be addressed:

1. In Figure 2b, the authors showed that transcripts with higher number of CDS m6A peaks tended to exhibit lower translation efficiency. I wonder whether the length of CDS might confound this observation. Longer CDS might have more m6A peaks and longer CDS is theoretically linked to lower translation efficiency. Testing the correlation of CDS m6A peak density and TE might be more appropriate.

2. The authors proposed that the RNA-helicase-containing reader YTHDC2 is involved in facilitating ribosome elongation along the m6A-modified CDS. (1) Is YTHDC2 itself present in the polysome fraction? (2) The helicase domain of the YTHDC2 was indicated to be important. Maybe the authors can demonstrate that using rescue experiments with WT and helicase-mutant DC2 in their reporter assay.

3. For Figure 4a, the sh-RNA treated cells were selected by puromycin, and then the cells were treated with the same reagent, puromycin, to label global protein synthesis. Please comment on whether the selection process may interfere with the protein synthesis quantification.

4. The m6A-mediated translation is quite complex. Readers in different cell lines operate differently. I wonder if the authors can analyze effects from 5' UTR, CDs, and 3' UTR with METTL3/14 KD in different cells (MEF, HEK and HeLa). Perhaps 3' UTR is more significant in HeLa and CDS more significant in MEF and HEK? This will be a very good point to add and expand a bit.

4. Some part of the manuscript can be improved for clearer information delivery, for example:

(1) It is not obvious how the distance to a motif is defined in Figure 2a. What does "when the 5'end of reads are counted" mean? The meaning of the bolded "A" in the triplet codons in the main text and figure legends was not defined.

(2) The construct of the dual luciferase reporter was not quite clear. Are the two luciferases expressed as an whole fusion protein, or there is an IRES structure or something similar in between the F-Luc and the hairpin structural motif so F-Luc and R-Luc are separate proteins? If it is the former, how shall "R-Luc/F-Luc" be interpreted? Please clarify.

(3) In the description of Figure S8f on page 10: the P values for CDS and 3'UTR groups are both way below 0.05 yet the former is described as "sensitive to"/"dramatically reduced upon" YTHDC2 depletion while the latter as "little effect". Please explain how the extent of DC2's effect was defined. Of course 0.05 is not a hard cut.

Reviewer #2 (Remarks to the Author):

The manuscript entitled „m6A in mRNA coding regions promotes translation via the RNA helicase-containing YTHDC2" by Mao and colleagues address the exciting question about the role of m6A within mRNAs and especially within the coding sequence. The authors provide a plethora of computational analyses that enable better (and unexpected) insights into the role of m6A during protein synthesis. The authors propose a stimulatory role of m6A on translation which, at least at first sight, contradicts (or extends) earlier finding by different groups working on m6A. So far m6A within CDSs were postulated to negatively impact translation elongation. However, they authors indirectly demonstrate that m6A can dissolve secondary structures and, through the assistance of YTHDC2, promote translation efficiency. To my opinion this, although somewhat counterintuitive, provides a novel and interesting aspect on the role of RNA modifications on gene expression. However, I think several points should be addressed.

Major remarks:

Page 4: The authors state that methylations at the 5' UTR do not correlate with TE. However on page 6 in it proposed that lower levels of m6A within the 5' UTR decrease ribosome occupancy. To me this seems contradictory. I would suppose that if the absence causes an decrease, the presence must be detectable as well.
Along this line, I wonder if that observed/described effect really is due to the stimulation of m6A on non-canonical translation initiation. I am surprised that these non-canonical events would be that abundant. Maybe the authors could comment on that. At this point I would suggest to insert the reference to the citations (Meyer&Jaffrey, Coots et al).

Page 5: The authors analyzed the nucleotide specific pattern of the methylation and correlated them to an increase of ribosome pausing and observed the largest impact of methylations when located at the 3rd codon position. In bacterial and eukaryotic system the effect of m6A on elongation was largest at the first nucleotide (demonstrated in the cited paper Choi et al. 2016 in bacteria and in Hoernes et al 2019 in HEK cells). I wonder about the methylations at the first nucleotide. It is shown without any comment SFig 3. I think this potential discrepancy is worth a sentence. Small side note: Already Choi et al showed a codon/sequence specific effect of m6A on translation. I would think it is not unexpected but rather expected.

Page 8: The authors used a dual luciferase assay to further strengthen their finding of a stimulator role of m6A on translation. I could not find any information on the design of their luciferase construct. I understand that the MALAT1 motif was introduced but I did the upstream Fluc gene have a stop codon or does the ribosome elongate through this motif or does Rluc start with the translation of this sequence? If it is translated it would be interesting to know at with codon position m6A is located. This would add another piece to the puzzle. It would be very helpful to get more detailed description of this experiment to better understand this biochemical assay.
Along this line: is there any "self-made" structural information that this motif is really altered by the methylation in the described setting and that the methylation is really there? I think it would add to the paper, if either the methylation or the structural changes are shown.

In 2005 the Noller lab published a paper (Takyar et al.; 2005) that shows a helicase activity of the ribosome dissolving rather long RNA/DNA helical regions. This was additionally shown by Tinoco lab (as cited in the manuscript) employing optical tweezers. Although this was shown in a bacterial system, I wonder why the eukaryotic ribosome should not be able dissolve these structural elements on its own? Are these structural elements that stable that the introduction of m6A as weak roadblock is easier to cope with?

If m6A in CDSs attract YTHDC2 and this protein interacts with XRN1, should there not be any indication of an altered stability of these mRNAs.

Minor points:

Supplementary Figure 3: There is now explanation for tAI. What does it stand for and what does it tell?

Page 3: In the following sentence it is stated: "However, neither the decoding feature of the endogenous mRNAs nor the…". I do not understand what the authors mean by "decoding feature"

Page 7: "(Fig 2d; bottom panel)" – I did not find the bottom panel

Page 11: typo: m6A-dependent (instead of dependenet; in line 4)

Supplementary Figure 6: The figure legend for 6b is confusing. It implicates that the GC content varies dependent on m6A.

Reviewer #3 (Remarks to the Author):

This is a very nice manuscript that documents the changes in translation and RNA structure as a consequence of m6A modifications in the mRNA coding sequence. One of the most important conclusions is the observation that contrary to what is expected, CDS methylation has a positive correlation on the translation efficiency of mRNAs. The authors suggest that by removing CDS m6A from methylated transcripts, translation efficiency can be further increased and that methylation appears to resolve mRNA structure. Hence, the manuscript explores a unique facet of CDS methylation on ribosome pausing and reveals the possibility that CDS m6A modifications prevent the formation of secondary structures. This is an important message, however with respect to the ribosome profiling and PARS analysis better representation of the data and further statistical analysis would greatly improve the message.

Major comments:

My main comments are on the analysis of Fig3 structure, which is central to the message of the paper and could be greatly improved. The in vitro PARS from extracted RNA (which contains m6a) in the supplement figure provides far better evidence for the author's conclusions then how they analyzed the icSHAPE data comparing in vivo vs in vitro. Are the MFE and GC content plots for CDS m6A sites in Figure 3? If so, please make this clear; if not, is this pattern specific to CDS m6A sites?

For Fig 3C, one suggestion is to try using windowed Gini index rather than raw reactivities. The icSHAPE signal looks wider distributed in the plot (as well as in the supplemental figure with in vitro icSHAPE data). This may actually indicate it's more structured, which is the opposite of author's conclusion from this plot. It does not argue against reduced structure due to m6A in CDS; it only reveals that without perturbing m6A levels and re-measuring structure, it is not possible to conclude on changes to the structure.

Along the same lines it would be very important to perhaps cross-reference and compare the Mettl3-/- vs WT mESC icSHAPE data in the original icSHAPE paper. It would be important and relatively easy to re-analyze that dataset around your CDS m6A peaks and confirm that reduced structure is still observed.

Overall, the Ribo-seq analysis is reasonably performed and my only concern is that the authors should

do a better job at making sure to have enough statistical power to provide a cut-off for detecting false positive Mettl3 sensitive genes. For the Ribo-seq data shown in Fig 2e and Fig 4d, the strengths of the conclusions being made would be greatly increased if the authors include replicates and false positive rates. This would enable a more accurate estimate to decide the cut-off for stratifying MFE distributions.

Reviewer #4 (Remarks to the Author):

In "m6A in mRNA coding regions promotes translation via the RNA helicase-containing YTHDC2", Mao et al. study the effects of m6A methylation in coding regions of mRNA. Evidence is provided that methylation is used to resolve local secondary structure by recruiting the helicase YTHDC2. This is potentially interesting to a broad audience. The manuscript reports ribosomal profiling data comparing cells lines deficient with methylation with wildtype.

Major Revisions:
The manuscript relies on ribosomal profiling data to interpret the extent of translation. Translation efficiency (TE), the normalized ribosomal density, is interpreted as the extent at which a message is translated. For example, the inverse correlation between TE and methylation is assumed to indicate inactive translation: "It is rather consistent with the notion that CDS methylation occurs on transcripts with relatively inactive translation [page 4]."
I am concerned that TE is not the clearest method for assessing the extent of translation. Initiation is likely as important as translation rate in determining the extent of translation [Philos Trans R Soc Lond B Biol Sci. 2017 372: 20160183]. Increased initiation will increase TE, but this manuscript does not draw and relationships between methylation and initiation. The relationship between translation rate and ribosome occupancy are probably the opposite that the manuscript assumes. For a given rate of translation initiation, slow or stalled ribosomes will increase TE but possibly reduce overall translation. Conversely, faster translation rates with constant initiation rates would lower TE, but also might not improve overall translation depending on whether initiation or elongation is rate limiting.
In my opinion, the manuscript should focus on pause sites that are related to methylation. Pausing can be compared across cell lines. Pausing can reduce overall translation depending on the rates of initiation and elongation.

Figure 3A shows the estimated folding free energy change for a local (30 nucleotide) sliding window of RNA sequence. It is interpreted as a low folding free energy change (stable structure) at methylation sites. But, the figure shows an abrupt increase in folding free energy change centered at the methylation site with local decreases in the 5′ and 3′ directions. This, of course, corresponds to the local decrease in GC fraction shown in panel B.
Overall, the local structure stability is not particularly convincing, especially given the increase in folding free energy change at the window centered on the exact methylation site. The folding free energy observation is highly correlated with GC fraction because GC pairs are more stable than AU or GU pairs. It is customary to consider folding stability as compared to controls with identical dinucleotide content, expressed as Z score (numbers of standard deviations from the mean). This indicates whether an RNA is locally organized to fold into stable structures. See, for example: Proc Natl Acad Sci U S A. 2005. 102:2454 or RNA. 2005. 11:578. The estimates of folding stability should be switched to z-scores of folding stability for dinucleotide-matched shuffled controls. Low Z-scores would be convincing local RNA secondary structures.

Minor Revisions:
On page 6, it is unclear what is meant by "While MEF cells with scramble control showed a prominent ribosome pausing at the methylated A site." Specifically, what "scramble" means is unclear.

On page 7, the best reference for “In particular, m6A installation destabilizes RNA secondary structures.” Is the study by Kierzek and Kierzek on folding stability [Nucleic Acids Res. 2003 31:4472]. This should be cited.
On page 7, “we first analyzed the structural potential predicted by ViennaRNA” cites reference 26, but should cite the Vienna package [Algorithms for Molecular Biology. 2011. 6:26].

Typos, etc.:
The text is generally clear and concise, but there are some typos and small errors:
On page 2, “Both the 5′ and 3′ untranslated regions (UTRs) bear many cis-acting elements that is intricately linked to the regulation of translation initiation.” Should be “Both the 5′ and 3′ untranslated regions (UTRs) bear many cis-acting elements that are intricately linked to the regulation of translation initiation.”
On page 2, “N6-methyladenosine (m6A) is the most abundant internal base modification occurred on eukaryotic mRNAs.” Should be “N6-methyladenosine (m6A) is the most abundant internal base modification occurring on eukaryotic mRNAs.”
On page 3, “YTHDF1 and YTHDF3 promotes cap-dependent mRNA translation” should be “YTHDF1 and YTHDF3 promote cap-dependent mRNA translation”.
On page 3, the sentence: “Our findings establish the physiological significance of
m6A methylation in CDS and uncovered non-overlapping function of m6A reader proteins.” is awkward.
On page 3, “To avoid false positives due to background noises” should be “To avoid false positives due to background noise”.
Om page 5, “Notably, transcripts harboring the conserved m6A sites exhibit significantly lower ribosome occupancy than the one containing the non-conserved sites.” Should be “Notably, transcripts harboring the conserved m6A sites exhibit significantly lower ribosome occupancy than ones containing the non-conserved sites.”
On page 5, “presence of m6A interferes the decoding process of ribosomes” should be “presence of m6A interferes with the decoding process of ribosomes”.
On page 8, “m6A-depedent” should be “m6A-dependent”.
On page 11, “m6A-dependenet” should be “m6A-dependent”.

We are pleased to receive the unanimous agreement from all the Referees that the unexpected role of CDS methylation in translation is *exciting*, *interesting*, *and important*. We have carefully considered all the Referees' concerns and thoroughly revised our manuscript accordingly. In particular, we have ***conducted rescue experiments using YTHDC2 mutants*** to validate the role of the helicase domain. We have also ***performed additional data analysis*** to strengthen the original conclusion. As a result, five supplementary figures have been updated. We sincerely hope that the Referees and the editor will find the revised manuscript suitable for publication in ***Nature Communications.***

A detailed point-by-point response to Referees' comments is listed below.

Referee #1:

In this manuscript, Mao et. al. investigated how m6A methylation at mRNA coding regions (CDS) modulates ribosome dynamics and translation. The authors reported that CDS m6A facilitates translation by opening up mRNA secondary structures, and suggested this process is mediated through the m6A reader YTHDC2 which contains RNA helicase domains.

This work contains new information very useful to the community. It addresses the regional effects of m6A and helps clarify the role of m6A on translational regulation. It also represents a good example of "correlation does not imply causation"—something many should keep in mind when interpreting functional significance of nucleic acid modifications from high-throughput sequencing data. I am in favor of publishing this work if the following comments can be addressed:

We are very pleased and grateful to receive the Referee's positive comments about the significance of our study.

1. In Figure 2b, the authors showed that transcripts with higher number of CDS m6A peaks tended to exhibit lower translation efficiency. I wonder whether the length of CDS might confound this observation. Longer CDS might have more m6A peaks and longer CDS is theoretically linked to lower translation efficiency. Testing the correlation of CDS m6A peak density and TE might be more appropriate.

We believe the Referee is referring Figure 1d instead of Figure 2b. In this regard, the Referee is absolutely correct that both translation efficiency (TE) and the number of m6A peaks can be influenced by the CDS length. To follow the Referee's suggestion, we stratified mRNAs based on CDS m6A peak density (or CDS m6A coverage). As shown in **Figure 1a** of this letter, the negative correlative between TE and the CDS m6A coverage support our original conclusion. Notably, the CDS m6A coverage is independent of the CDS length.

To further factor out the CDS length as well as the sequence-associated variation, we conducted an independent analysis by comparing the distribution of TE for transcripts with or without CDS methylation. For the m6A(–) group bearing non-methylated RRAC, transcripts with high TE (top 5%) and low TE (bottom 5%) have similar fractions (**Figure 1b** in this letter). By contrast, the m6A(+) group bearing

methylated RRAC is highly enriched in the low TE group (>19 fold). This result suggests that it is the m6A modification but not the sequence *per se* that correlates with TE. In the revised manuscript, we have added these new analytical results into the Supplementary Figure 2b and 2c and described them in the main text.

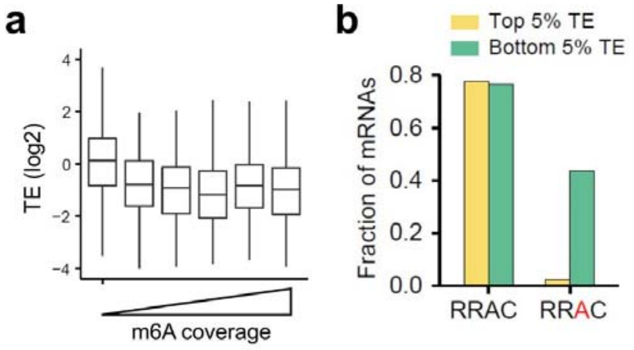

Figure 1 Correlation of CDS m6A and translation efficiency. **(a)** A box plot shows the relative translation efficiency for transcripts with different mRNA coverage normalized by RNA abundance. **(b)** Transcripts with top and bottom 5% TE were selected followed by grouping based on CDS methylation. mRNAs with at least one m6A consensus motif in CDS (RRAC) or CDS m6A peak (RRAC) were counted, respectively.

Page 4: The similar finding was observed by analyzing CDS m^{6}A coverage (Supplementary Fig. 2b), which is further corroborated by the distribution of TE for transcripts with or without CDS m^{6}A (Supplementary Fig. 2c).

2. The authors proposed that the RNA-helicase-containing reader YTHDC2 is involved in facilitating ribosome elongation along the m6A-modified CDS. (1) Is YTHDC2 itself present in the polysome fraction? (2) The helicase domain of the YTHDC2 was indicated to be important. Maybe the authors can demonstrate that using rescue experiments with WT and helicase-mutant DC2 in their reporter assay.

The Referee raised two excellent points regarding YTHDC2. We have followed the Referee's experimental suggestion by first examining the distribution of YTHDC2 in polysome fractions separated by sucrose gradients. We found that YTHDC2 is enriched in the monosome instead of polysome fractions (**Figure 2** of this letter). This is rather expected because YTHDC2-targeted mRNAs are of low translation efficiency. By contrast, mRNAs with high translation efficiency are generally enriched in polysomes. Notably, this result is consistent with the previous report (Hsu *et al*. 2017 Cell Research).

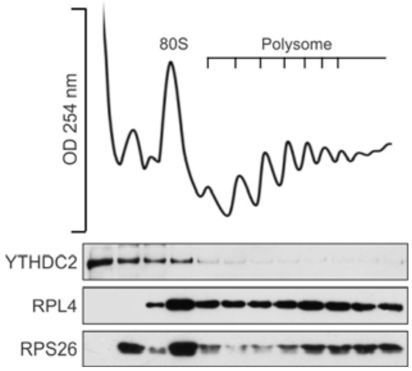

Figure 2 Polysome profiling of HEK293 cells followed by western blotting of fractions using antibodies indicated.

With respect to the rescue experiment, we completely agree with the Referee that it is important to use a helicase-mutant YTHDC2 together with the wild type. To create a helicase-dead mutant, we introduced E332Q mutation into the conserved RecA domain of a cloned human YTHDC2. The resultant mutant lacks the helicase activity as reported by a previous study (Wojtas *et al*. 2017 Mo Cell). In HEK293 cells lacking endogenous YTHDC2, we added back either wild type or E332Q mutant via plasmid transfection (**Figure 3a** of this letter). Luciferase reporter assay showed that only the wild type YTHDC2, but not the helicase-dead mutant, was able to restore the Rluc/Fluc

ratio (**Figure 3b** of this letter). This new result is now presented in Supplementary Figure 9 of the revised manuscript.

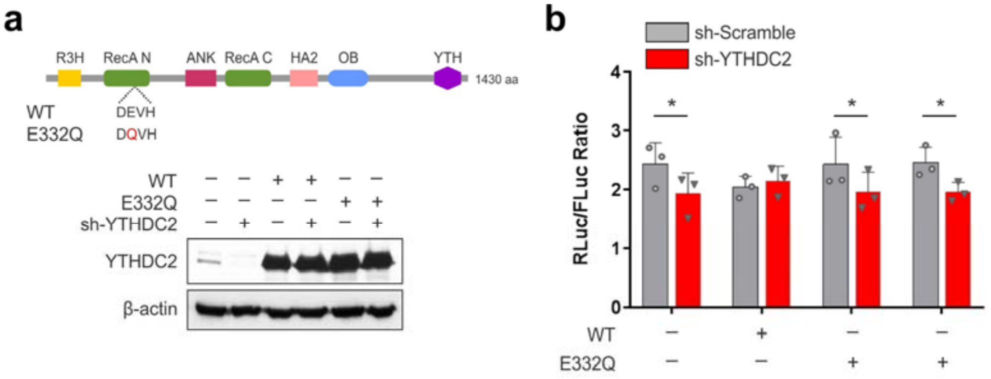

Figure 3 **(a)** Schematic view of human YTHDC2 domain structures with the helicase domain RecA highlighted in wild type (WT) and E332Q mutant. The bottom panel shows the expression levels of exogenous human YTHDC2 (WT and E332Q) in HEK293 cells with YTHDC2 knockdown. **(b)** HEK293 cells with or without YTHDC2 knockdown were added back WT YTHDC2 or the E332Q mutant together with the plasmid expressing the dual luciferase reporter. The Rluc/Fluc ratio is shown after normalization to the Scramble control. Error bars, mean ± s.e.m.; Single-tailed t-test. * $P < 0.05$.

Page 11: To confirm the critical role of helicase activity, we conducted a rescue experiment by introducing an E332Q mutation to inactivate the helicase of YTHDC2 (Supplementary Fig. 9a). Luciferase reporter assay showed that only the wild type YTHDC2, but not the helicase-dead mutant, was able to restore the Rluc/Fluc ratio (Supplementary Fig. 9b).

3. For Figure 4a, the sh-RNA treated cells were selected by puromycin, and then the cells were treated with the same reagent, puromycin, to label global protein synthesis. Please comment on whether the selection process may interfere with the protein synthesis quantification.

The Referee brought up an astute point in terms of puromycin selection and labeling. We are fully aware of the fact that shRNA knockdown cells are selected by puromycin. As a result, the puromycin labeling efficiency in vivo is reduced in these cells. However, puromycin incorporation occurs efficiently in cell lysates as demonstrated by previous reports (David *et al*. 2012 J Cell Biol). In fact, it is the labeling in cell lysates that reflects the true amount of ribosomes associated with mRNAs. Regardless, the Referee's concern is highly relevant and we routinely included the scramble control that was equally selected by puromycin. Therefore, we are confident that different puromycin signals represent changes of global protein synthesis. We thank the Referee for understanding.

4. The m6A-mediated translation is quite complex. Readers in different cell lines operate differently. I wonder if the authors can analyze effects from 5' UTR, CDs, and 3' UTR with METTL3/14 KD in different cells (MEF, HEK and HeLa). Perhaps 3' UTR is more significant in HeLa and CDS more significant in MEF and HEK? This will be a very good point to add and expand a bit.

The Reviewer's suggestion is well-taken. We analyzed several published data sets in different cell lines, including HeLa, HEK293, ESC, and EB cells. Upon METTL3 knockdown, there is a general reduction of translation efficiency for m6A(+) transcripts regardless of the methylated regions (**Figure 4** of this letter). This is rather expected because METTL3 silencing globally reduces mRNA methylation. This result also implies that mRNA methylation positively regulates translation in general, which is in line with the previous studies about the positive role of YTHDF1 and YTHDF3 in translation. Transcripts with CDS m6A modifications, however, appear to be more sensitive to the silence of METTL3 than other regional methylation. This result further supports the notion that CDS methylation positively regulates translation.

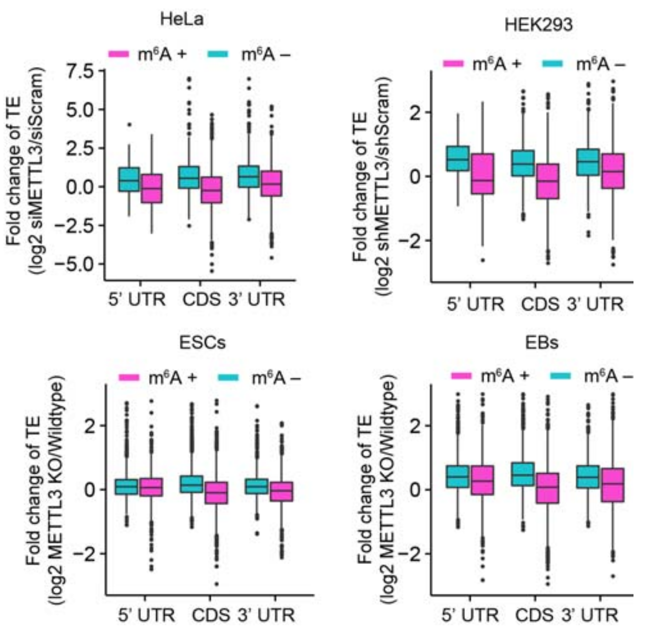

Figure 4 Transcripts are stratified based on regional methylation in the form of m6A). The fold change of translation efficiency upon METTL3 knockdown is plotted as boxplots for mRNAs bearing regional methylation (m6A +) or not (m6A –).

5. Some part of the manuscript can be improved for clearer information delivery, for example:

(1) It is not obvious how the distance to a motif is defined in Figure 2a. What does "when the 5'end of reads are counted" mean? The meaning of the bolded "A" in the triplet codons in the main text and figure legends was not defined.

We appreciate the Referee's obvious care in reviewing our manuscript. In Figure 2a, the ribosome density was aggregated along mRNA regions aligned to the RRAC motif with the position of A defined as "0". Since ribosome footprints are of varied length, we chose the 5' end of footprints to count the amount of reads at particular positions. In the main text and figure legends, the bolded **A** means a modification at this position. We have clarified this issue in the revised manuscript by using the red **A** to keep the consistency.

(2) The construct of the dual luciferase reporter was not quite clear. Are the two luciferases expressed as an whole fusion protein, or there is an IRES structure or something similar in between the F-Luc and the hairpin structural motif so F-Luc and R-Luc are separate proteins? If it is the former, how shall "R-Luc/F-Luc" be interpreted? Please clarify.

We apologize for not being clearer in the original description of the vector construction. We intended to create a reporter mimicking CDS methylation by fusing Fluc and Rluc. Instead of using an IRES element, we inserted a sequence derived from *Malat1* as the

methylation at A2577 modulates the secondary structure (Liu *et al*. 2015 Nature). As a result, the full length fusion protein contains both Fluc and Rluc activities. The *Malat1* structural motif is expected to hinder ribosome elongation depending on the methylation status. The partially synthesized protein gives rise Fluc activity but not Rluc. Therefore, the ratio of Rluc/Fluc serves as a good indicator of CDS structures. In the revised manuscript, we have clarified this confusion in the main text, figure legends, as well as the methods.

(3) In the description of Figure S8f on page 10: the P values for CDS and 3'UTR groups are both way below 0.05 yet the former is described as "sensitive to"/"dramatically reduced upon" YTHDC2 depletion while the latter as "little effect". Please explain how the extent of DC2's effect was defined. Of course 0.05 is not a hard cut.

We appreciate the Reviewer's attention to detail. As pointed out by the Referee, both CDS and 3' UTR groups show significantly reduced TE upon YTHDC2 knockdown (Wilcox-test, all P values < 0.05). However, it is also clear that the reduced TE is more significant in CDS group than other groups. The "little effect" in the original text was meant for 5'UTR. We agree with the Referee that is not a proper term in this context. In the revised manuscript, we have rephrased the statement to avoid possible confusion.

> **Page 10:** By comparing the TE of transcripts with or without CDS methylation, we found that mRNAs bearing methylated CDS are more sensitive to YTHDC2 depletion than the one with 3'UTR methylation (Fig. 4c). Notably, YTHDC2 knockdown has little effect on the translation of mRNAs with 5' UTR methylation (Supplementary Fig. 8f), confirming the regional effect of YTHDC2.

Referee #2:

The manuscript entitled „m6A in mRNA coding regions promotes translation via the RNA helicase-containing YTHDC2" by Mao and colleagues address the exciting question about the role of m6A within mRNAs and especially within the coding sequence. The authors provide a plethora of computational analyses that enable better (and unexpected) insights into the role of m6A during protein synthesis. The authors propose a stimulatory role of m6A on translation which, at least at first sight, contradicts (or extends) earlier finding by different groups working on m6A. So far m6A within CDSs were postulated to negatively impact translation elongation. However, they authors indirectly demonstrate that m6A can dissolve secondary structures and, through the assistance of YTHDC2, promote translation efficiency. To my opinion this, although somewhat counterintuitive, provides a novel and interesting aspect on the role of RNA modifications on gene expression. However, I think several points should be addressed.

We thank the Reviewer for concisely summarizing our work. We are very pleased to receive the Reviewer's positive comments about the novelty and the significance of our study.

Major remarks:

1. Page 4: The authors state that methylations at the 5' UTR do not correlate with TE. However on page 6 in it proposed that lower levels of m6A within the 5' UTR decrease

ribosome occupancy. To me this seems contradictory. I would suppose that if the absence causes an decrease, the presence must be detectable as well.

Along this line, I wonder if that observed/described effect really is due to the stimulation of m6A on non-canonical translation initiation. I am surprised that these non-canonical events would be that abundant. Maybe the authors could comment on that. At this point I would suggest to insert the reference to the citations (Meyer&Jaffrey, Coots et al).

We appreciate the Referee's insightful comments. There might be some confusion between Figure 1c and Supplementary Figure 4b, partly due to the lack of clarity in the original description. In Figure 1c, the lack of correlation between 5'UTR methylation and TE is among different transcripts, whereas the decreased TE in Supplementary Figure 4b refers to the same transcript under different 5'UTR methylation. On the other words, 5'UTR methylation is not the sole determinant of TE but has the stimulatory effect for the same transcript. This is consistent with our previous findings that 5'UTR m6A promotes non-canonical translation initiation when the canonical translation is suppressed (Zhou *et al*. 2018 Mol Cell; Meyer *et al.* 2015 Cell).

Nevertheless, we agree with the Referee that the stimulatory effect of 5' UTR methylation cannot be solely explained by non-canonical translation initiation. Notably, m6A readers like YTHDF1 and YTHDF3 appear to promote translation initiation via canonical mechanisms like mRNA looping (Wang *et al*. 2015 Cell). <u>We have further clarified the correlation issue in the revised manuscript and cited the references mentioned above.</u>

2. Page 5: The authors analyzed the nucleotide specific pattern of the methylation and correlated them to an increase of ribosome pausing and observed the largest impact of methylations when located at the 3rd codon position. In bacterial and eukaryotic system the effect of m6A on elongation was largest at the first nucleotide (demonstrated in the cited paper Choi et al. 2016 in bacteria and in Hoernes et al 2019 in HEK cells). I wonder about the methylations at the first nucleotide. It is shown without any comment SFig 3. I think this potential discrepancy is worth a sentence. Small side note: Already Choi et al showed a codon/sequence specific effect of m6A on translation. I would think it is not unexpected but rather expected.

The Referee raised an important comment regarding the correlation between nucleotide-specific methylation and ribosome pausing. Notably, both Choi *et al.* and Hoernes *et al.* used an artificial system to test the effect of modification. Since m6A is preferentially deposited to the adenine within the RRACH motif, the first and second positions of codon AAA are unlikely to be methylated in vivo. We therefore believe it is important to investigate m6A-mediated ribosomal pausing using Ribo-seq data, which reflect translation of endogenous mRNAs. It is worth mentioning that although GAA shows much stronger pausing than GAC, our data do not necessarily indicate that the third position modification has more significant effect than other positions. As mentioned in the main text, the same m6A position in both AGA and GGA codons is only associated with a slight increase of ribosome density (Supplementary Fig. 3c). Therefore, we conclude that m6A-mediated ribosome pausing is more likely codon-specific rather than nucleotide position-specific. In the revised manuscript, <u>we have further clarified this issue</u>.

3. Page 8: The authors used a dual luciferase assay to further strengthen their finding of a stimulator role of m6A on translation. I could not find any information on the design of their luciferase construct. I understand that the MALAT1 motif was introduced but I did the upstream Fluc gene have a stop codon or does the ribosome elongate through this motif or does Rluc start with the translation of this sequence? If it is translated it would be interesting to know at with codon position m6A is located. This would add another piece to the puzzle. It would be very helpful to get more detailed description of this experiment to better understand this biochemical assay.

Along this line: is there any "self-made" structural information that this motif is really altered by the methylation in the described setting and that the methylation is really there? I think it would add to the paper, if either the methylation or the structural changes are shown.

We apologize for not being clearer in the original description of the luciferase reporter construction. We intended to create a reporter mimicking CDS methylation by fusing Fluc and Rluc. Therefore, the stop codon of Fluc have been removed in order to generate a fusion protein. We inserted in-between a sequence derived from *Malat1* as the methylation at A2577 modulates the secondary structure (Liu *et al*. 2015 Nature). From the encoded amino acid sequence, the m6A at A2577 happens to be within the codon of ACT. In the revised manuscript, we have added more detailed information in the construction of dual luciferase reporters.

As the luciferase reporter is totally "home-made", we agree with the Referee that it is important to demonstrate that the inserted *Malat1* sequence exhibits the same feature as the endogenous *Malat1*. For the structural aspect, we have shown that the presence of the *Malat1* structural motif hinders ribosome elongation, resulting in reduced translation of downstream Rluc. To address the Referee's concern about the methylation status of the inserted sequence, we employed a method called "SELECT" to achieve site-specific detection of m6A (Xiao *et al*. 2018 Angew Chem Int Ed Engl). The SELECT method exploits the ability of m6A to hinder the single-base elongation activity of DNA polymerases and the nick ligation efficiency of ligases. We designed site-specific probes for A2577 of human *Malat1* that fortunately differs from the mouse counterpart. It is gratifying that the inserted *Malat1* sequence showed reduced ligation products upon METTL3 knockdown and increased ligation products after knocking down the m6A demethylase FTO (**Figure 5** of this letter). Importantly, the A2577G mutant does not show significance changes. These results correspond to the behavior of endogenous *Malat1*, supporting its unambiguous methylation. We have now included these new results in the revised manuscript as Supplementary Figure 7a. We hope the Referee will agree that this new piece of data strengthens our original conclusion.

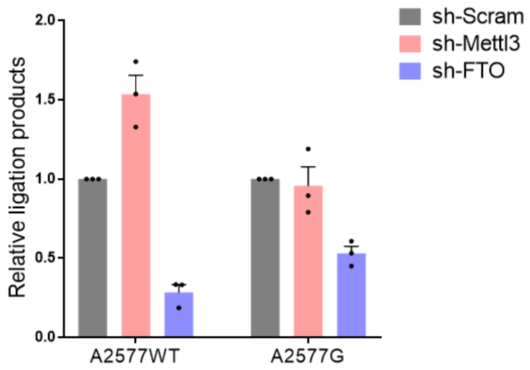

Figure 5 Measurement of m6A levels on A2577 of *Malat1* in MEF cells transfected with luciferase reporters with WT or A2577G mutation. Notably, lowered ligation products indicate higher m6A levels. Data are presented as mean ± s.e.m. from three replicates. *, $p < 0.05$.

4. In 2005 the Noller lab published a paper (Takyar et al.; 2005) that shows a helicase activity of the ribosome dissolving rather long RNA/DNA helical regions. This was additionally shown by Tinoco lab (as cited in the manuscript) employing optical tweezers. Although this was shown in a bacterial system, I wonder why the eukaryotic ribosome should not be able dissolve these structural elements on its own? Are these structural elements that stable that the introduction of m6A as weak roadblock is easier to cope with?

The Referee brought up an astute point regarding the helicase activity of ribosomes. RNA molecule can fold into numerous local secondary structures, with large variations in structural stability (Mustoe *et al*. 2018). We agree that the ribosome itself can act as a helicase to unfold the majority of stem-loop structures within CDS. However, as shown by Qu *et al*. 2011, the stability of secondary structure affects the unfolding efficiency quite significantly. Ribosomes could move very slowly in regions with extremely stable structures, which may cause ribosome accumulation or even trigger translation quality control. As demonstrated by Doma and Parker 2006, a stable stem-loop structure results in ribosomal stalling, which are subsequently recognized and targeted for endonucleolytic cleavage.

Interestingly, mRNA regions with long and stable structures tend to be methylated in the form of m6A. Our data suggest that m6A modification destabilizes these structures, thereby facilitating ribosome movement along structural regions. This mechanism is expected to reduce the probability of ribosomal stalling. Further supporting this notion, Kretschmer *et al*. 2018 reported direct interaction between the YTHDC2 and the small ribosomal subunit. This finding implies a possible cooperation between different types of helicases to unwind structures in methylated regions. Due to the speculative nature, we did not expand this notion further in the current manuscript.

5. If m6A in CDSs attract YTHDC2 and this protein interacts with XRN1, should there not be any indication of an altered stability of these mRNAs.

The Referee brought up an important point, which is highly relevant to our current study. To investigate the effect of YTHDC2 on RNA stability, we took advantage of the published data sets derived from mouse testes (Bailey *et al.* 2017 eLife). We classified mRNAs into YTHDC2 targets and non-targets based on fRIP-seq in mouse Testes (Bailey *et al.* 2017 eLife). Upon YTHDC2 knockdown, we observed an increase of abundance of the targeted mRNAs (**Figure 6** of this letter). This result suggests that YTHDC2 could decrease RNA stability, which is consistent with previous studies (Hsu et al. 2017, Wojtas et al. 2017). However, this finding is not contradictory to the positive role of YTHDC2 in translation. In our study, the translation efficiency (TE) is computed after normalization with the mRNA abundance.

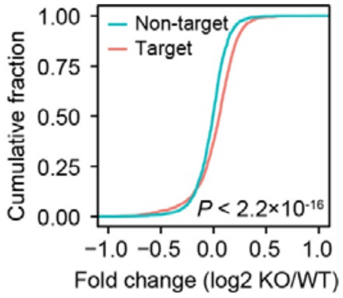

Figure 6 All mRNAs were separated into YTHDC2 target and non-target groups. The fold changes of RNA levels upon YTHDC2 knockdown were compared (Wilcxon-test, $P < 2.2\times10^{-16}$).

Minor points:

1. Supplementary Figure 3: There is now explanation for tAI. What does it stand for and what does it tell?

We appreciate the Referee's effort to improve our manuscript. The tRNA adaptation index (tAI) is a measure of the tRNA usage by coding sequences. tAI is commonly used to measure codon optimality, which is expected to affect ribosome elongation rate. The results in Supplementary Figure 3b and 3c suggest that the codon optimality has limited effect on the pausing at the methylation sites. In the revised manuscript, we have added more details on tAI calculation.

2. Page 3: In the following sentence it is stated: "However, neither the decoding feature of the endogenous mRNAs nor the…". I do not understand what the authors mean by "decoding feature"

We apologize for the confusion here. Since the single molecule assay by Choi *et al* 2016 is based on the *in vitro* system, we were meant to say "it remains unclear how the methylated codon is decoded inside cells". We have revised this sentence in the revised manuscript.

> **Page 3:** However, neither the decoding feature of methylated codons within endogenous mRNAs nor the physiological significance of CDS methylation has been clearly defined.

3. Page 7: "(Fig 2d; bottom panel)" – I did not find the bottom panel

We thank the Referee for pointing out this error, which was supposed to be Fig. 2c. We are glad to have this opportunity to fix this error in the revised manuscript.

4. Page 11: typo: m6A-dependent (instead of dependenet; in line 4)

We thank the Referee for careful reading of our manuscript. We have corrected this typo in the revised manuscript.

5. Supplementary Figure 6: The figure legend for 6b is confusing. It implicates that the GC content varies dependent on m6A.

We appreciate the Referee's obvious care in reviewing our manuscript. Supplementary Figure 6b indicates a higher GC-content in the regions flanking the m6A site but not the methylated site itself. This is consistent with the finding that m6A occurs on the loop rather than the stem of RNA structures. We have revised the legend in the revised manuscript.

Referee #3:

This is a very nice manuscript that documents the changes in translation and RNA structure as a consequence of m6A modifications in the mRNA coding sequence. One of the most important conclusions is the observation that contrary to what is expected, CDS methylation has a positive correlation on the translation efficiency of mRNAs. The authors suggest that by removing CDS m6A from methylated transcripts, translation efficiency can be further increased and that methylation appears to resolve mRNA structure. Hence, the manuscript explores a unique facet of CDS methylation on ribosome pausing and reveals the possibility that CDS m6A modifications prevent the formation of secondary structures. This is an important message, however with respect to the ribosome profiling and PARS analysis better representation of the data and further statistical analysis would greatly improve the message.

We thank the Reviewer for concisely summarizing our work. We are very pleased to receive the Reviewer's positive comments about the novelty and the significance of our study.

Major comments:

1. My main comments are on the analysis of Fig3 structure, which is central to the message of the paper and could be greatly improved. The in vitro PARS from extracted RNA (which contains m6a) in the supplement figure provides far better evidence for the author's conclusions then how they analyzed the icSHAPE data comparing in vivo vs in vitro. Are the MFE and GC content plots for CDS m6A sites in Figure 3? If so, please make this clear; if not, is this pattern specific to CDS m6A sites?

For Fig 3C, one suggestion is to try using windowed Gini index rather than raw reactivities. The icSHAPE signal looks wider distributed in the plot (as well as in the supplemental figure with in vitro icSHAPE data). This may actually indicate it's more structured, which is the opposite of author's conclusion from this plot. It does not argue against reduced structure due to m6A in CDS; it only reveals that without perturbing m6A levels and re-measuring structure, it is not possible to conclude on changes to the structure.

We appreciate the Referee's insightful comments. We have made it clear that Figure 3a and 3b show the MFE and GC content plotted for CDS m6A sites. In Figure 3c, the large variation of icSHAPE signals of methylated regions is likely due to the less total number of m6A sites than the number of non-methylated RRAC motif. Nevertheless, we agree that the Gini index could provide additional structural information around m6A sites. As shown in **Figure 7** in this letter, the Gini index shows the similar pattern as the icSHAPE signals. Methylated regions tend to exhibit more secondary structures *in vitro* as evidenced by the higher Gini index. By contrast, the same methylated regions show reduced Gini index *in vivo*, an indication of resolved structures. Regardless, we agree with the Referee that these analysis cannot conclude dynamic structural changes. In the revised manuscript, we have toned down our conclusion to avoid overstatement. Additionally, we have included the analysis of Gini index as Supplementary Fig. 6a – 6b and described them in the main text.

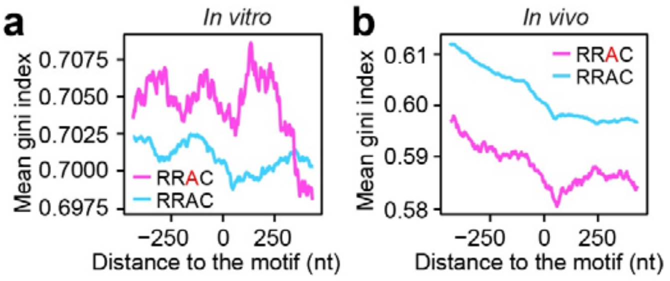

Figure 7 Comparison of Gini index of methylated and non-methylated regions. **(a)** The Gini index of *in vitro* icSHAPE signals is plotted along mRNA regions surrounding the RRAC motif with (pink) or without (blue) m^6A modification. **(b)** The Gini index of *in vivo* icSHAPE signals around methylated and non-methylated regions. Notably, a low *Gini index* indicates a less structured region.

2. Along the same lines it would be very important to perhaps cross-reference and compare the Mettl3-/- vs WT mESC icSHAPE data in the original icSHAPE paper. It would be important and relatively easy to re-analyze that dataset around your CDS m6A peaks and confirm that reduced structure is still observed.

We have followed the Referee's suggestion by reanalyzing the icSHAPE data derived from Mettl3-/- and WT cells respectively. We grouped mRNAs into m6A(+) and m6A(-) based on CDS methylation followed by comparison of fold changes of icSHAPE signals after METTL3 knockout. We observed a quite significant decrease (p = 0.0001) of icSHAPE signals for mRNAs with CDS m6A (**Figure 8** in the letter). Therefore, CDS m6A methylation appears to resolve mRNA secondary structures *in vivo*. We have now presented this analysis result as Supplementary Figure 7c in the revised manuscript.

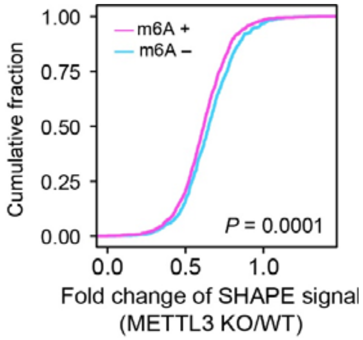

Figure 8 Using the icSHAPE data sets derived from mESC cells, the fold change of icSHAPE signals upon METTL3 knockout is plotted as accumulative fractions for mRNAs bearing CDS methylation (m6A +) or not (m6A –).

3. Overall, the Ribo-seq analysis is reasonably performed and my only concern is that the authors should do a better job at making sure to have enough statistical power to provide a cut-off for detecting false positive Mettl3 sensitive genes. For the Ribo-seq data shown in Fig 2e and Fig 4d, the strengths of the conclusions being made would be greatly increased if the authors include replicates and false positive rates. This would enable a more accurate estimate to decide the cut-off for stratifying MFE distributions.

We appreciate the Referee's effort to improve our manuscript. For METTL3 and YTHDC2 knockdown, we have two biological replicates of Ribo-seq for each condition. To address the Referee's concern about false positive rates, we conducted a simulation assay in order to find mRNA regions with increased ribosome footprints upon METTL3 or YTHDC2 knockdown. This way would allow us to detect METTL3 sensitive genes by controlling for the false positive rates. Under fold change >2 and FDR < 0.05, while the number of identified pausing regions is reduced (5,836 and 4,194 for METTL3 and YTHDC2 respectively), we still found a significantly lower MFE in identified pausing regions (**Figure 9** of this letter). We believe (and hope the Referee would concur) that our conclusion is based on sound statistical power.

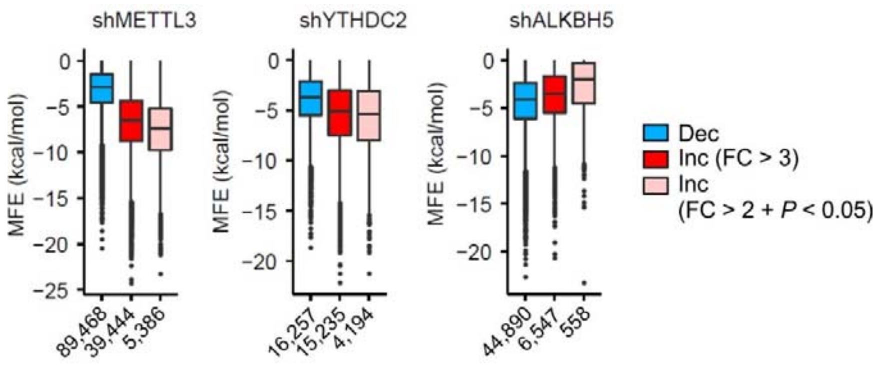

Figure 9 The regions with ribosomal pausing were determined based on different statistical criteria. In comparison to the negative control with decreased ribosome density, pausing regions identified by >3 fold change or simulation method (fold change >2 and FDR < 0.05) are comparable.

Referee #4:

In "m6A in mRNA coding regions promotes translation via the RNA helicase-containing YTHDC2", Mao et al. study the effects of m6A methylation in coding regions of mRNA. Evidence is provided that methylation is used to resolve local secondary structure by recruiting the helicase YTHDC2. This is potentially <u>*interesting*</u> *to a broad audience. The manuscript reports ribosomal profiling data comparing cells lines deficient with methylation with wildtype.*

We thank the Referee for concisely summarizing our work. We are pleased to receive the Referee's positive comments that our study is interesting to a broad audience.

Major Revisions:

The manuscript relies on ribosomal profiling data to interpret the extent of translation. Translation efficiency (TE), the normalized ribosomal density, is interpreted as the extent at which a message is translated. For example, the inverse correlation between TE and methylation is assumed to indicate inactive translation: "It is rather consistent with the notion that CDS methylation occurs on transcripts with relatively inactive translation [page 4]."

I am concerned that TE is not the clearest method for assessing the extent of translation. Initiation is likely as important as translation rate in determining the extent of translation [Philos Trans R Soc Lond B Biol Sci. 2017 372: 20160183]. Increased initiation will increase TE, but this manuscript does not draw and relationships between methylation and initiation. The relationship between translation rate and ribosome occupancy are probably the opposite that the manuscript assumes. For a given rate of translation initiation, slow or stalled ribosomes will increase TE but possibly reduce overall translation. Conversely, faster translation rates with constant initiation rates would lower TE, but also might not improve overall translation depending on whether initiation or elongation is rate limiting.

In my opinion, the manuscript should focus on pause sites that are related to methylation. Pausing can be compared across cell lines. Pausing can reduce overall translation depending on the rates of initiation and elongation.

The Referee is absolutely correct in terms of the interpretation of translation efficiency (TE). We completely agree that TE can be affected by both initiation and elongation. In

the current study, we used both computational and experimental approaches to assess the translational status. For TE calculation, we minimized the variation of initiation rates by separating mRNAs based on regional methylation (Figure 1c). In particular, the CDS m6A group has negligible 5'UTR methylation. Importantly, we relied on independent approaches to validate the effect of CDS methylation on translational output. The dual luciferase assay effectively factors out the influence of initiation.

As suggested by the Referee, we have focused on ribosome pausing sites related to CDS methylation. We found ribosomal accumulation in methylated regions after removing CDS methylation by METTL3 knockdown (Figure 2c and 2d). By taking advantage of RNA structural data sets, we provide evidence of structural changes related to CDS m6A. In the revised manuscript, we have paid attention to the proper interpretation of TE.

Figure 3A shows the estimated folding free energy change for a local (30 nucleotide) sliding window of RNA sequence. It is interpreted as a low folding free energy change (stable structure) at methylation sites. But, the figure shows an abrupt increase in folding free energy change centered at the methylation site with local decreases in the 5' and 3' directions. This, of course, corresponds to the local decrease in GC fraction shown in panel B.

Overall, the local structure stability is not particularly convincing, especially given the increase in folding free energy change at the window centered on the exact methylation site. The folding free energy observation is highly correlated with GC fraction because GC pairs are more stable than AU or GU pairs. It is customary to consider folding stability as compared to controls with identical dinucleotide content, expressed as Z score (numbers of standard deviations from the mean). This indicates whether an RNA is locally organized to fold into stable structures. See, for example: Proc Natl Acad Sci U S A. 2005. 102:2454 or RNA. 2005. 11:578. The estimates of folding stability should be switched to z-scores of folding stability for dinucleotide-matched shuffled controls. Low Z-scores would be convincing local RNA secondary structures.

We appreciate the Referee's effort to improve our manuscript. In Figure 3a, the abrupt increase of MFE peak at the methylate site is due to the same sequence motif we used for alignment. It is expected to be comparable between methylated and non-methylated regions. The striking difference is mainly found at flanking regions, an indication of broad structure formation. The Referee's suggestion about z-score analysis is instructive. We generated random sequences while keeping dinucleotide content (Altschul and Erickson. 1985 Mol Biol Evol), followed by z-score calculation based on the previous study (Clote et al. 2005 RNA). Similar to the MFE distribution, we found lowered z-score values flanking the m6A sites (**Figure 10** of this letter). Once again, the central peak at the methylation site maintains for the same reason mentioned above.

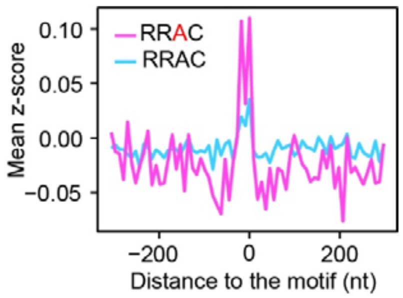

Figure 10 The z-score of mean folding energy (MFE) around the m6A sites was calculated by using a sliding window with 30 nt in length and a step of 3nt. For each sequence, 30 random sequences were generated by shuffling nucleotides while keeping dinucleotide content.

Importantly, our structural interrogation is not limited to the sequence-based analysis. In fact, it is the discrepancy between sequence-based prediction and *in vivo* structural mapping (icSHAPE and PARS) promoted us to propose that local m6A resolves mRNA secondary structures. In the revised manuscript, we have further clarified this confusion in the main text.

Minor Revisions:

On page 6, it is unclear what is meant by "While MEF cells with scramble control showed a prominent ribosome pausing at the methylated A site." Specifically, what "scramble" means is unclear.

We apologize for not being clearer in the original description. The scramble control refers to the MEF cells transfected with shRNA with scrambled non-targeting sequence, serving as control for shRNA targeting METTL3. In the revised manuscript, we have used "scramble shRNA control" in the main text.

On page 7, the best reference for "In particular, m6A installation destabilizes RNA secondary structures." Is the study by Kierzek and Kierzek on folding stability [Nucleic Acids Res. 2003 31:4472]. This should be cited.

We thank the Referee for alerting us of this reference. We have added this citation in the revised manuscript.

On page 7, "we first analyzed the structural potential predicted by ViennaRNA" cites reference 26, but should cite the Vienna package [Algorithms for Molecular Biology. 2011. 6:26].

We thank the Referee for careful reading of our manuscript. We have corrected this citation in the revised manuscript.

Typos, etc.:

The text is generally clear and concise, but there are some typos and small errors:

On page 2, "Both the 5' and 3' untranslated regions (UTRs) bear many cis-acting elements that is intricately linked to the regulation of translation initiation." Should be "Both the 5' and 3' untranslated regions (UTRs) bear many cis-acting elements that are intricately linked to the regulation of translation initiation."

On page 2, "N6-methyladenosine (m6A) is the most abundant internal base modification occurred on eukaryotic mRNAs." Should be "N6-methyladenosine (m6A) is the most abundant internal base modification occurring on eukaryotic mRNAs."

On page 3, "YTHDF1 and YTHDF3 promotes cap-dependent mRNA translation" should be "YTHDF1 and YTHDF3 promote cap-dependent mRNA translation".

On page 3, the sentence: "Our findings establish the physiological significance of m6A methylation in CDS and uncovered non-overlapping function of m6A reader proteins." is awkward.

On page 3, “To avoid false positives due to background noises” should be “To avoid false positives due to background noise”.

Om page 5, “Notably, transcripts harboring the conserved m6A sites exhibit significantly lower ribosome occupancy than the one containing the non-conserved sites.” Should be “Notably, transcripts harboring the conserved m6A sites exhibit significantly lower ribosome occupancy than ones containing the non-conserved sites.”

On page 5, “presence of m6A interferes the decoding process of ribosomes” should be “presence of m6A interferes with the decoding process of ribosomes”.

On page 8, “m6A-depedent” should be “m6A-dependent”.

On page 11, “m6A-dependenet” should be “m6A-dependent”.

We appreciate the Referee obvious care in reviewing our manuscript. We are glad to have this opportunity to fix all the typos. We have also carefully checked the entire manuscript to ensure error-free.

Closing words

We again thank all the Referees for their time in reading our manuscript and providing expert commentary. We have thoroughly revised our manuscript and addressed all of the concerns. In particular, we have performed additional experiments to strengthen our original conclusions. We believe the revised manuscript has been much improved. We wish to convey our most sincere thanks to all the Referees and the Editor for considering our work for publication in ***Nature Communications***.

Reviewers' comments:

Reviewer #1 (Remarks to the Author):

The authors have addressed all my comments. This is a very nice work.

If I may I want to bring up another point: The role of m6A in translation promotion is very much context dependent. In HEK cells YTHDF1 does not seem to affect translation at all. The authors want to point out the context and stimulation-mediated translation upregulation. The last part besides HEK it would be nice to verify in HeLa or at least point out the important context dependency. In systems that YTHDF1 and YTHDF3 may not play roles in translation YTHDC2 may have more important roles.

In addition, the subcellular localization of YTHDC2 should be studied and discussed.

Reviewer #2 (Remarks to the Author):

All points raised were addressed appropriately by the authors. It is a really interesting mansucript!

Reviewer #3 (Remarks to the Author):

The reviewers have carried out the additional analysis that I have requested. I feel that this manuscript is now ready for publication.

Reviewer #4 (Remarks to the Author):

The authors provided a careful and convincing rebuttal of my concerns. On the other hand, they were not responsive with making changes to the manuscript. The text is almost entirely identical to the previous version. The figures are unchanged. Below I detail my ongoing concerns.

My primary concern previously was the reliance on translation efficiency (TE) as the measure of total translation. (In particular, my concern is that ribosome occupancy is a function of translation rate and initiation. Assuming higher TE indicates higher amounts of translation is incorrect when pausing rates are variable.) The authors' rebuttal, however, is correct; the dual luciferase assay provides strong experimental evidence for their conclusions. The rebuttal also points out that separating mRNAs by region of methylation helps to mitigate the concern about variation in initiation. The manuscript was not revised to point this out; other readers might also appreciate some acknowledgement that initiation rates are also important to consider.

For the RNA folding stability, the abrupt increase in folding free energy change is stated in the rebuttal to be caused by the position of the motif (RRAC) being aligned. But does the position 0 mean that the motif is at the 5' end of the window, or the middle of the window? Some additional information in the caption and/or methods would be helpful. If 0 means the motif is at the 5' end, then the window is presumably disrupting the structure centered at the methylation site (i.e. it is an artifact of having a window). If 0 means the methylation site is the middle of the 30 nucleotide window, I am still concerned that there is less potential for stable folding right at the methylation site.
The z-score plot also shows a small, but mostly consistent effect for greater folding stability. There is, however, no obvious single motif being formed (which would appear as a significant z score; $z < -2$). This suggests a non-specific effect of increased GC content. It would be helpful for the z score plot to be provided to readers (in the Supplement would be adequate.)

My minor concerns were all addressed with changes to the manuscript.

I have some additional suggestions. On page 5, the RRAC sequence motif is first mentioned (line 136). It might be helpful for some readers to also state this is the consensus sequence with the m6A methylation site.
On page 8, line 212, "cores" should be "scores". On line 229, "the two base pairs GU to GC" would be better as "the two GU base pairs to GC".

We are pleased to receive the unanimous agreement from all the Referees that our revised manuscript has been significantly improved. We have thoroughly addressed all the remaining concerns with a detailed point-by-point response listed below.

Referee #1:
The authors have addressed all my comments. This is a very nice work.

If I may I want to bring up another point: The role of m6A in translation promotion is very much context dependent. In HEK cells YTHDF1 does not seem to affect translation at all. The authors want to point out the context and stimulation-mediated translation upregulation. The last part besides HEK it would be nice to verify in HeLa or at least point out the important context dependency. In systems that YTHDF1 and YTHDF3 may not play roles in translation YTHDC2 may have more important roles.

In addition, the subcellular localization of YTHDC2 should be studied and discussed.

We agree with the Referee that the role of m^{6}A in mRNA translation could be context dependent. We have clarified this point in the discussion of the revised manuscript. We have also followed the Referee's experimental suggestion by examining the subcellular localization of YTHDC2 in different cell lines. To this end, we have conducted immuno-fluorescence staining of HEK293, HeLa and MEF cells using a YTHDF2 antibody. Indeed, we observed that the subcellular localization of YTHDC2 varies across different cell lines (**Figure 1a** in this letter). In particular, MEF cells show predominant cytoplasmic signals of YTHDC2. In human cells however, YTHDC2 can be found in both cytoplasm and nucleus. In parallel, we also examined the subcellular localization of YTHDF1, which shows strong cytoplasmic signals as reported previously (**Figure 1b** in this letter). Collectively, these results further emphasize the importance of the context in considering the function of YTHDC2. We have added this new result as supplementary figure 10 in the revised manuscript.

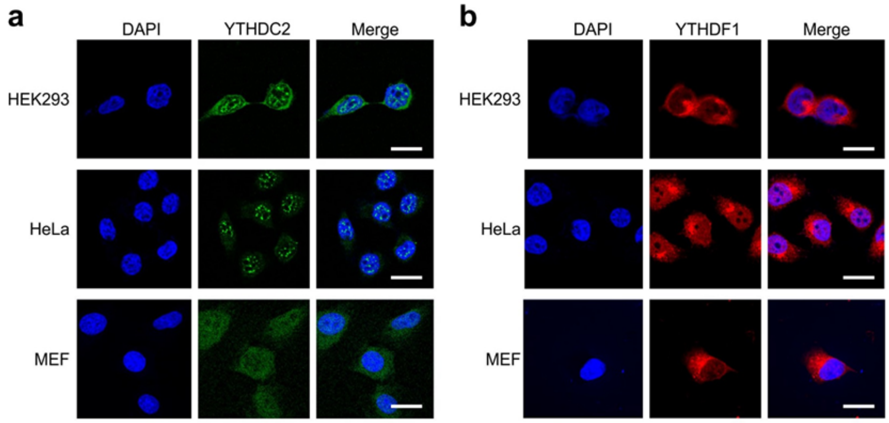

Figure 1 Subcellular localization of YTHDC2 **(a)** and YTHDF1 **(b)** in different cell lines. Endogenous YTHDC2 and YTHDF1 in HEK293, HeLa, and MEF cells were Immuno-stained using antibodies. Nuclei was counter-stained with DAPI.

Page 13: Akin to the tissue-specific expression of YTHDC2, different cell types have varied subcellular localizations of m^{6}A readers (Supplementary Fig. 10). Therefore, the functionality of m^{6}A reader is likely context-dependent.

Referee #4:

The authors provided a careful and convincing rebuttal of my concerns. On the other hand, they were not responsive with making changes to the manuscript. The text is almost entirely identical to the previous version. The figures are unchanged. Below I detail my ongoing concerns.

We apologize for not highlighting the changes in our original manuscript, even though we did make substantial amount of revision in the main text. To fully address the Referee's remaining concerns, we have not only highlighted all the changes in the newly revised manuscript, but also listed the revised text below.

1. My primary concern previously was the reliance on translation efficiency (TE) as the measure of total translation. (In particular, my concern is that ribosome occupancy is a function of translation rate and initiation. Assuming higher TE indicates higher amounts of translation is incorrect when pausing rates are variable.) The authors' rebuttal, however, is correct; the dual luciferase assay provides strong experimental evidence for their conclusions. The rebuttal also points out that separating mRNAs by region of methylation helps to mitigate the concern about variation in initiation. The manuscript was not revised to point this out; other readers might also appreciate some acknowledgement that initiation rates are also important to consider.

We completely agree with the Referee that the TE values can be influenced by varied initiation and elongation rates, especially in case of ribosome pausing. We have further clarified this issue in the revised manuscript.

> **Page 8:** As ribosome occupancy can be influenced by both initiation and elongation, potential pausing sites in CDS could increase the ribosome density with reduced translational output. To directly demonstrate the critical role of m^{6}A in CDS structures and subsequent translational outcomes, we constructed a fusion reporter by inserting a structural motif between firefly luciferase (Fluc) and renilla luciferase (Rluc) (Fig. 3d).

2. For the RNA folding stability, the abrupt increase in folding free energy change is stated in the rebuttal to be caused by the position of the motif (RRAC) being aligned. But does the position 0 mean that the motif is at the 5' end of the window, or the middle of the window? Some additional information in the caption and/or methods would be helpful. If 0 means the motif is at the 5' end, then the window is presumably disrupting the structure centered at the methylation site (i.e. it is an artifact of having a window). If 0 means the methylation site is the middle of the 30 nucleotide window, I am still concerned that there is less potential for stable folding right at the methylation site.

We apologize for not being clearer in the original description of Fig. 3a. "0" means the methylation site is the middle of the window. Based on the folding free energy around m^{6}A sites, the Referee is correct that the downstream region of m^{6}A sites (>250 nt) have less potential for stable structures when compared to the upstream region. However, both upstream and downstream regions around the aligned m^{6}A sites showed dramatically reduced free energy than the non-methylated counterpart. We believe this data clearly support the notion that there is an increased structural potential in the flanking regions around m^{6}A sites. In the revised manuscript, we have updated the legend of Fig. 3a.

3. The z-score plot also shows a small, but mostly consistent effect for greater folding stability. There is, however, no obvious single motif being formed (which would appear as a significant z score; z < -2). This suggests a non-specific effect of increased GC content. It would be helpful for the z score plot to be provided to readers (in the Supplement would be adequate.).

We agree with the Reviewer that there is no single motif (or a consensus motif) in the flank regions of m^6A sites, based on z-score value. In addition, we did not find any consensus structural motif in the flank regions using RNAalifold. This is quite expected because such structures would act as physical barriers to impede ribosome movement. We have followed the Referee's suggestion by including the z score plot in the revised manuscript as Supplementary Fig. 6a.

4. My minor concerns were all addressed with changes to the manuscript.

I have some additional suggestions. On page 5, the RRAC sequence motif is first mentioned (line 136). It might be helpful for some readers to also state this is the consensus sequence with the m6A methylation site.

We appreciate the Referee's obvious care in reviewing our manuscript. We have revised the sentence in the newly revised manuscript.

> **Page 5:** This position corresponds to the methylated codon at the ribosomal A site. As a negative control, we used the same RRAC sequence motif but without methylation.

5. On page 8, line 212, "cores" should be "scores". On line 229, "the two base pairs GU to GC" would be better as "the two GU base pairs to GC".

We thank the Referee for alerting us of these errors. We are glad to have this opportunity to fix these typos in the revised manuscript.
